# Comparative genome analysis unravels pathogenicity of *Xanthomonas albilineans* causing sugarcane leaf scald disease

**DOI:** 10.1186/s12864-022-08900-2

**Published:** 2022-09-26

**Authors:** MeiLin Li, YiXue Bao, YiSha Li, Sehrish Akbar, GuangYue Wu, JinXia Du, Ronghui Wen, Baoshan Chen, MuQing Zhang

**Affiliations:** State Key Laboratory of Conservation and Utilization for Subtropical Agri-Biological Resources & Guangxi Key Laboratory for Sugarcane Biology, Guangxi University, Nanning, 530005 Guangxi China

**Keywords:** Sugarcane leaf scald disease, *Xanthomonas albilineans* (*Xal*), *Xanthomonas sacchari* (*Xsa*), Two-component system, Single Nucleotide Polymorphism (SNP)

## Abstract

**Background:**

*Xanthomonas* is a genus of gram-negative bacterium containing more than 35 species. Among these pathogenic species, *Xanthomonas albilineans* (*Xal*) is of global interest, responsible for leaf scald disease in sugarcane. Another notable *Xanthomonas* species is *Xanthomonas sachari* (*Xsa*), a sugarcane-associated agent of chlorotic streak disease.

**Result:**

The virulence of 24 *Xanthomonas* strains was evaluated by disease index (DI) and Area Under Disease Progress Curve (AUDPC) in the susceptible inoculated plants (GT 46) and clustered into three groups of five highly potent, seven mild virulent, and twelve weak virulent strains. The highly potent strain (*X. albilineans*, *Xal* JG43) and its weak virulent related strain (*X. sacchari*, *Xsa* DD13) were sequenced, assembled, and annotated in the circular genomes. The genomic size of JG43 was smaller than that of DD13. Both strains (JG43 and DD13) lacked a Type III secretory system (T3SS) and T6SS. However, JG43 possessed Salmonella pathogenicity island-1 (SPI-1). More pathogen-host interaction (PHI) genes and virulent factors in 17 genomic islands (GIs) were detected in JG43, among which six were related to pathogenicity. Albicidin and a two-component system associated with virulence were also detected in JG43. Furthermore, 23 *Xanthomonas* strains were sequenced and classified into three categories based on Single Nucleotide Polymorphism (SNP) mutation loci and pathogenicity, using JG43 as a reference genome. Transitions were dominant SNP mutations, while structural variation (SV) is frequent intrachromosomal rearrangement (ITX). Two essential genes (*rpfC*/*rpfG*) of the two-component system and another gene related to SNP were mutated to understand their virulence effect. The mutation of *rpfG* resulted in a decrease in pathogenicity.

**Conclusion:**

These findings revealed virulence of 24 *Xanthomonas* strains and variations by 23 *Xanthomonas* strains. We sequenced, assembled, and annotated the circular genomes of *Xal* JG43 and *Xsa* DD13, identifying diversity detected by pathogenic factors and systems. Furthermore, complete genomic sequences and sequenced data will provide a theoretical basis for identifying pathogenic factors responsible for sugarcane leaf scald disease.

**Supplementary Information:**

The online version contains supplementary material available at 10.1186/s12864-022-08900-2.

## Background

*Xanthomonas* (from the Greek Xanthos, meaning 'yellow,' and monas, meaning ‘entity’) is a large genus of gram-negative, yellow-pigmented bacteria associated with plants. The genus, which locates at the base of the Gamma proteobacteria, comprises 27 species that cause severe diseases in ~ 400 plant hosts, including a wide variety of economically important crops, such as rice, citrus, banana, cabbage, tomato, pepper, and bean [1–4]. Pathogenic species and pathovars have a high degree of host plant and tissue specificity and invade either the xylem elements of the vascular system or the intercellular spaces of the mesophyll parenchyma tissue.

Functional and comparative genomic studies clarify how this group of bacteria has adapted to exploit an extraordinary diversity of plant hosts and host tissues. An improved understanding of the pathogenic adaptations of *Xanthomonas* spp. will promote the development of much-needed improvements in the prevention and control of plant bacterial disease. In the following sections, we discuss how functional and comparative genomics shed light on the pathogenicity, adaptation, and evolution of *Xanthomonas spp*. The complete genome sequences of 11 *Xanthomonas* strains have been determined to date.

Sugarcane leaf scald is a bacterial disease caused by *Xanthomonas albilineans* (*Xal*). The disease was observed in various sugarcane-growing countries [5, 6]. Sugarcane leaf scald, one of the quarantine diseases in China, was initially identified in Beihai (Guangxi) in 2016 and quickly spread through all sugarcane growing areas in Yunnan, Guangdong, Zhejiang, and Fujian [7–9]. *Xal*, a gram-negative capsular bacterium with rod-shaped pilin, has a diameter of (0.6–1.0 μm) × (0.2–0.3 μm) micron and an optimal culturing temperature of 25℃-28℃ [10]. *Xal* can spread from root to root, leaf to leaf, and through the air [11, 12]. When *Xal* colonizes the sugarcane xylem, it systemically spreads to the entire host plant, causing a significant reduction in sugarcane yield and economic loss to the sugarcane industry [5, 13]. During a disease survey in Guangxi, another related pathogen, *X. sacchari* (*Xsa*), was reported to cause chlorotic streak disease of sugarcane in China [14]. The *Xsa* infected sugarcane leaves (cv. ROC22) showed chlorotic white streak symptoms ("pencil lines"), which were extended longitudinally from base to tip of the leaves [14].

The pathogenic factors of *Xanthomonas* include exopolysaccharides and biofilms [15, 16]. Compared with other *Xanthomonas* genomes, *Xal* does not produce xanthan gum and lacks a T3SS responsible for transmitting effector proteins or virulent factors to induce the immunological responses in the host [17–19]. However, *Xal* produces a toxin called albicidin, a small molecule synthesized by nonribosomal peptide synthases (NRPSs) [20–22]. Albicidin is a DNA gyrase inhibitor with different structures from other DNA helicases [23, 24]. This toxin prevents chloroplast formation, causing leaves to turn white [17, 24, 25]. However, mutant strains still caused virulence when the albicidin-related genes were knocked out, indicating that albicidin is not the primary factor influencing the prevalence of *Xal* [17].

Quorum sensing (QS) of the two-component system is a language of intercellular communication to induce a specific physiological response in *Xanthomonas* [26, 27]. A gene cluster of regulation pathogenic factor (*rpf*) is involved in mediating extracellular polysaccharides, biofilms, and motility [28]. The virulence of *Xanthomonas* decreased when the *rpf* gene was knocked out [29, 30]. *RpfF* produces a Diffusible Signal Factor (DSF) compound, a medium-chain fatty acid (FA) diffusible signal factor. When *rpfF* produces DSF, the *rpfC* gene senses DSF for autophosphorylation and transmits the signal to *rpfG* for regulating the expression of disease-related genes [28, 30]. *RpfG* controls the expression of pathogenic factors by mediating the concentration of Cyclic-di–GMP [28]. To explore the pathogenicity associated with genomic features of *X. albilineans*, we sequenced and assembled the genomes of two pathogenic strains; *X. albilineans* (JG43) and its related strain *X. sacchari* (DD13). Additionally, the other 23 strains of *X. albilineans* with varying virulent degrees were sequenced. The comparative genomic analysis was carried out to decipher the pathogenicity associated with the two-component system.

## Results

### Genomic features of *X. albilineans* JG43 and its related *X. sacchari* DD13

*X. albilineans* is the causal agent of sugarcane leaf scald, whereas *X. sacchari*, a related strain of *Xal*, causes sugarcane leaf chlorotic streak disease in China (Fig. S1, Table S1). The genomes of *Xal* JG43 were sequenced at 220.96 × coverage in long reads and 377.34 × coverage in short reads using Oxford Nanopore Technology (ONT) and Illumina Hi-Seq Technology*.* In contrast, *Xsa* DD13 was sequenced at 294.37 × coverage in long reads using the SMRT sequencing technology of Pacific Biosciences.

**Fig. 1:**
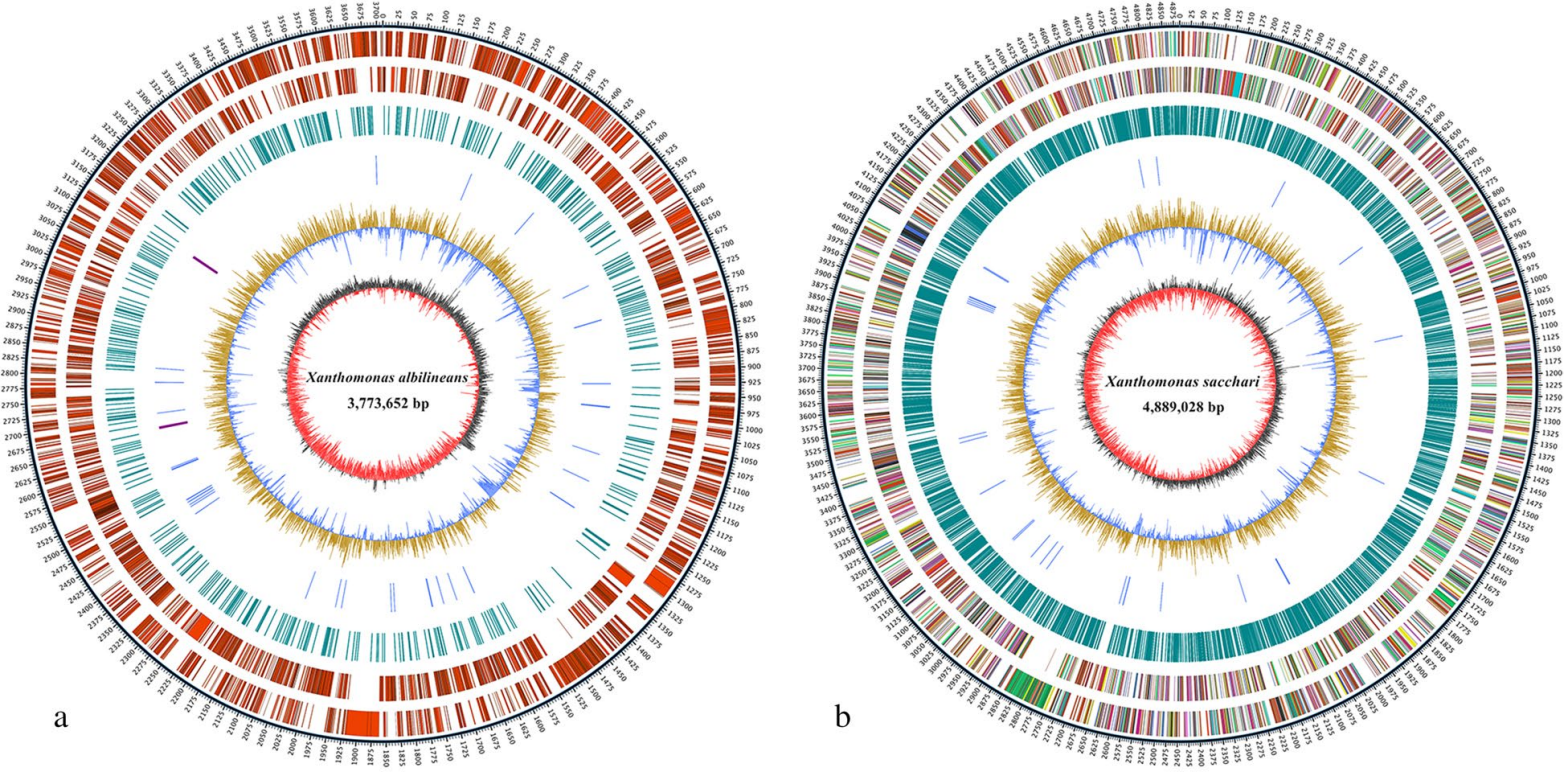
Circular representation of the *Xanthomonas*.** A**: genomic features of *X. albilineans* JG43. **B**: genomic features of *X. sacchari* DD13. The outermost (1^st^) circle was labeled with the size of the genome, each scale measuring 5 KB; The 2^nd^ and 3^rd^ circles were genes on the positive and negative chains of the genome, respectively, and different colors represent different COG functional classifications. The 4^th^ circle was a repeat sequence; The 5^th^ circle was tRNA and rRNA, blue is tRNA, purple is rRNA; The 6^th^ circle was GC content. The light-yellow part indicated that the GC content in this region was higher than the average GC content in the genome. The higher the peak value was, the greater the difference was with the average GC content. The blue part indicated that the GC content in this region was lower than the average GC content in the genome. The innermost circle was GC-SKEW, where dark gray represented the region with G content greater than C and red represented the region with C content greater than G

The circular genome of *Xal* JG43 was assembled at 3.77 MB with 62.98% of GC content and 0.61% of the repetitive sequence (Fig. 1a; Table 1). However, the genome of *Xsa* DD13 consisted of one circular chromosome at the size of 4.88 MB with 69.61% of GC and 2.99% of repeat sequences (Fig. 1b; Table 1). The short interspersed nuclear elements (SINE), long interspersed nuclear elements (LINE), and small RNA and DNA elements were only detected in the DD13 genome. However, only 0.01% of unclassified repeat elements were found in the JG43 strain (Table S2). JG43 genome contained 3,124 predicted genes with an average gene length of 1,057 bp, whereas DD13 had 4,022 ones with 1,052 bp at an average size (Table 2). JG43 had seven pseudogenes and one plasmid. The plasmid included all *VirB* gene clusters (VirB1, VirB5, VirB6, VirB8, VirB9, VirB10) of T4SS and RelE/ParE family toxin of the type II toxin-antitoxin system (Table S3). However, DD13 lacked pseudogenes and plasmids. The DD13 strain had more CAZys than JG43, including 95 glycoside hydrolases (GHs), 50 glycosyltransferases (GTs), 28 carbohydrate-binding modules (CBMs), 52 carbohydrate esterase (CE), 5 polysaccharide lyases (PLs), and 12 auxiliary activities (AA) (Table S4). Compared to DD13, more pathogen-host interaction (PHI) genes and virulent factors were predicted from the virulent factor database (VFDB) in JG43 (Table 3). VFDB mainly concentrates on the effector delivery system, type IV pili (T4P) of adherence, immune modulation, flagella of motility, metabolic factor, and exotoxin. Bsa T3SS, Rickettsiales vir homolog (Rvh) T4SS, and Trw T4SS protein of effector delivery system were found only in JG43. Rvh T4SS functions to replicate genes whose components are distributed throughout the genome [31]. Trw is unique in T4SSs, which is necessary for the cloning and colonization of bacteria [32]. However, (type IV Aeromonas pilus) Tap T4P was available in DD13, but nothing is known regarding the function of Tap T4P [33]. Syringopeptin in JG43 is a necrosis-inducing phytotoxin as a virulence determinant in the plant-pathogen interaction [34]. However, only cytolysin was detected in DD13. The capsular polysaccharide of Immune modulation in JG43 is a virulent factor inhibiting complement-mediated killing in bacteria [35]. In contrast, the HemO cluster of metabolic factors in DD13 can efficiently utilize heme in *Acinetobacter baumannii* [36] (Table S5). Genomic analysis revealed that pathogenicity-related genes of *Xal* JG43 were mainly concentrated on genomic islands, whereas the pathogenicity-related genes were involved in prophages in *Xsa* DD13. *Xal* JG43 had 17 genomic islands and 2 prophages, whereas DD13 had only 6 prophages and 3 genomic islands (Table 1). Blast analysis indicated that 6 of the 17 GIs in JG43 were associated with pathogenicity (Table S6). In JG43, genomic islands #1, #3, and #7 were comprised of genes associated with the Type IV secretion system (T4SS), including VirB2 to VirB11, which transfer either DNA or large proteins from one cell to another in eukaryotic or prokaryotic organisms [37–39]. Genes in genomic island # 10 were related to the export apparatus protein of the Type III secretory system (T3SS). Genomic Island # 12 was related to transcription factors or proteins associated with the two-component system, which help the bacteria adapt to their environment [40]. Genomic Island # 17 contained a type II toxin-antitoxin system.

**Table 1:** Genomic features of *X. albilineans* JG43 and its related *X. sacchari* DD13

	*X. albilineans* JG43	*X. sacchari* DD13
Gene total length (bp)	3,304,008	4,234,767
Plasmid length (bp)	63,110	-
Contig number	2	1
Pseudogene	7	0
Gene average (bp)	1,057	1,052
GI number	17	3
GI total length (bp)	282,390	40,858
GI average length (bp)	16,611	13,619
Prophage number	2	6
Prophage total length (bp)	66,801	185,126
Prophage average length (bp)	33,400	30,854
Coverage by repeats (bp)	22,980	146,007
Coverage by repeats (% of repeats)	0.61	2.99

GI: Genomic island

**Table 2:** Comparative genomics features of six *Xanthomonas* species

Genomic features	*Xal* JG43	*Xal* FJ1	*Xsa* DD13	*Xac* 306	*Xoo* PXO99A	*Xcc* 8004
Genome size (bp)	3,773,652	3,756,117	4,889,028	5,274,174	5,240,075	5,148,708
GC content (%)	62.98	63	69.61	64.7	64	65
Predict genes (in total)	3,124	3,167	4,022	4,533	5,083	4,462
CDS (in total)	3,117	3,083	4,022	4,434	4,666	4,309
Average CDS length (bp)	1,057	1,016	1,052	1,032	1,074	1,023
plasmid	1	1	0	2	0	0
tRNA	53	51	34	53	55	54
rRNA operons	6	6	6	6	6	6
Gene family number	2,903	2,866	3,252	3,717	3,036	3,581
Unique gene families	13	4	19	16	38	13
Unique genes	29	8	48	34	244	27
Disease	Sugarcane leaf scald	Sugarcane leaf scald	Chlorotic streak	Asiatic citrus canker	Bacterial blight of rice	Black rot of crucifers
Albicidin	+	+	-	-	-	-
Secretory system	I-II, IV-V	I-II, IV-V	I-II, IV-V	I-VI	I-III, V-VI	I-V
Colonization	Xylem	Xylem	Mesophyll	mesophyll tissue	Xylem	mesophyll tissue

Note:“ + ” present; “-” absent

**Table 3:** Statistics of function database and database annotation of *X. albilineans* JG43 and its related *X. sacchari* DD13

Database	*X. albilineans* JG43	*X. sacchari* DD13
GO	2,368 (75.80%)	2,637 (65.56%)
KEGG	1,716 (54.93%)	2,114 (52.56%)
Pfam	2,609 (83.51%)	3,469 (86.25%)
Swiss-Prot	1,859 (59.51%)	2,594 (64.50%)
TREMBL	1,859 (59.51%)	3,908 (97.17%)
NR	3,057 (97.86%)	3,961 (98.48%)
CAZy	154 (4.93%)	242 (6.02%)
CARD	2 (0.06%)	1 (0.02%)
PHI	1,081 (34.60%)	735 (18.27%)
VFDB	656 (21.00%)	468 (11.64%)
SP	397 (12.71%)	498 (12.38%)

Note: CAZy: Carbohydrate-active enzymes database; CARD: Comprehensive Antibiotic Research Database; PHI: Pathogen Host Interactions Database; VFDB: virulence factor database; SP: Secreted protein

Most pathogenic genes were available in prophages but not in the genomic island of *Xsa* DD13. All three genomic islands of DD13 were involved in Prophage #1. Genomic islands #1 were comprised of genes associated with histidine kinase. Genomic island #2 comprised of genes related to ATP-dependent endonuclease of the OLD family, while Genomic Island #3 had genes related to ATP-dependent Clp protease proteolytic subunit. Prophage #2 in *Xsa* DD13 was predicted to be associated with the Type II secretion system protein (T2SS) and T4SS pilis, which plays a vital role in the survival, and environmental adaption of pathogens [41], bacterial-host interaction, motility, and pathogenicity [39]. The genes in prophage #5 were related to the pathogenic locus and DNA-binding protein, delivering toxins and hydrolases to the cell surface of the gram-negative bacteria by T2SS. The gene encoded aminoglycoside phosphotransferase in Prophage #6 phosphorylates all aminoglycoside antibiotics, which aids in drug resistance in bacteria [42].

### Comparative genomic analysis of *Xanthomonas*

**Fig. 2:**
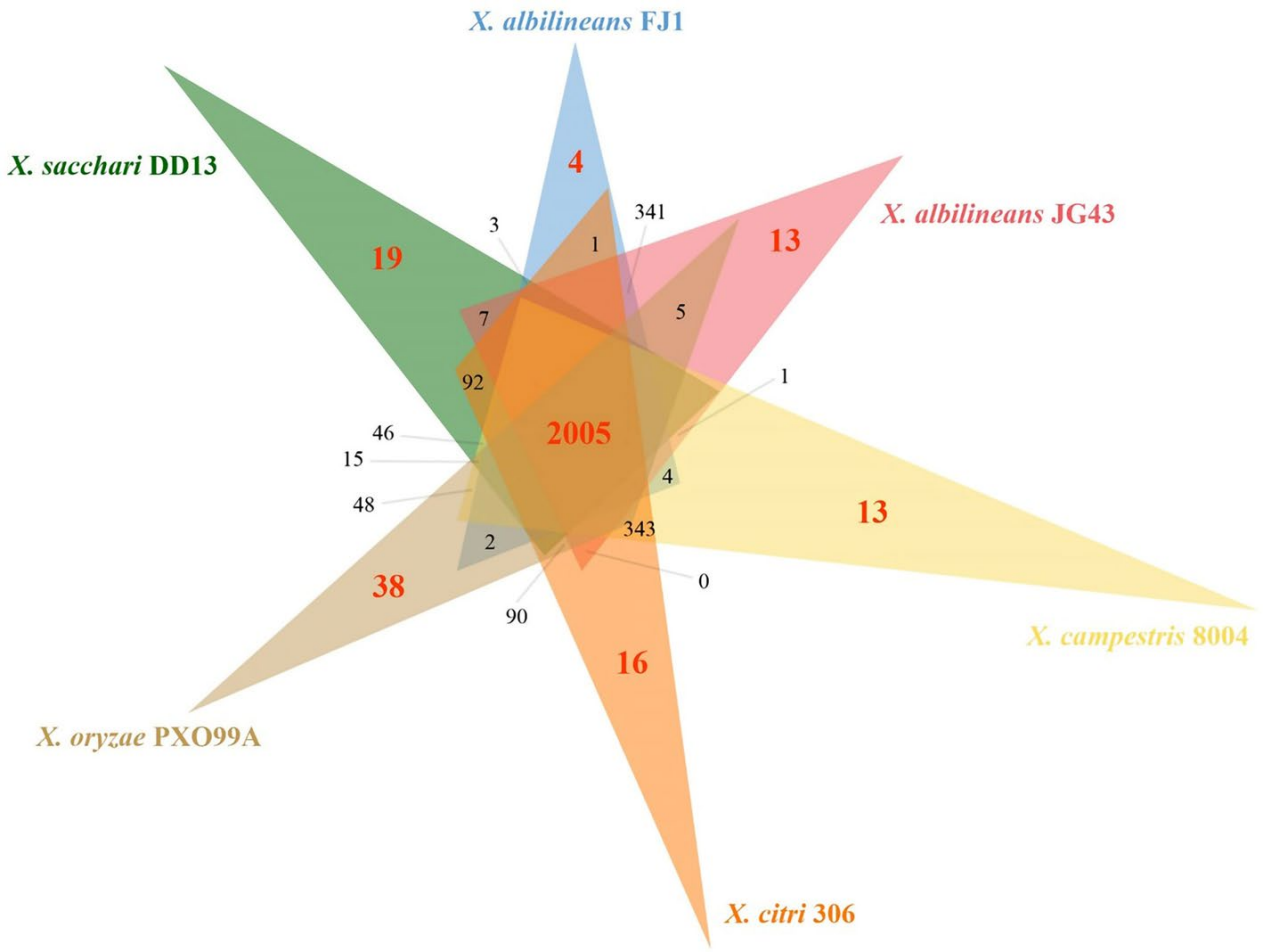
Venn diagrams showing predicted genes as determined by Orthologous clusters analyses among strains of *Xanthomonadaceae****.**** (i) X. albilineans* JG43 *(Xal* JG43*), X. albilineans* FJ1 (*Xal* FJ1), *X.sacchari* DD13 (*Xsa* DD13), *and X. citri pv. citri str.* 306 *(Xac* 306*), X. oryzae pv. oryzae* PXO99A (*Xoo* PXO99A), *X. campestris pv. campestris* 8004 (*Xcc* 8004)

The genomic features of the *Xal* JG43 were compared to the other five *Xanthomonadaceae* strains using OrthoMCL, including *Xsa* DD13, *Xal* FJ1 [19], *Xanthomonas citri pv.citri* strain 306 (*Xac* 306) [43], *Xanthomonas oryzae pv. oryzae* PXO99A (*Xoo* PXO99A) [44], and *Xanthomonas campestris pv. campestris* 8004 (*Xcc* 8004) [45]. A total of 2005 “core” orthologous proteins or coding DNA sequences were shared in all six *Xanthomonas*. Unique homologous genes were predicted in six strains, including 13 in *Xal* JG43, 19 in *Xsa* DD13, 4 in *Xal* FJ1, 38 in *Xoo* PXO99A, 14 in *Xac* 306, and 13 in *Xcc* 8004 (Fig. 2; Table 2). Ortholog comparisons identified 341 CDS specific to *Xal* JG43 and *Xal* FJ1 and fewer than 10 CDS of *Xal* JG43 specific to the other strains. The genome size of *Xal* JG43 was reduced by about 1.4 Mb compared with *Xac* 306, *Xoo* PXO99A, and *Xcc* 8004 (Table 2). The number of genes was reduced in *Xal* JG43 and *Xal* FJ1, including 898 in DD13, 1,409 in *Xac* 306, 1,959 in *Xoo* PXO99A, and 1,338 in *Xcc* 8004. Genomic sequence data identified only one plasmid in *Xal* JG43 and *Xal* FJ1 and two plasmids (pXAC33 and pXAC64) in *Xac* 306, while no plasmid was detected in *Xsa* DD13, *respectively Xoo* PXO99A and *Xcc* 8004.

### Type III Secretory System (T3SS)

T3SS is controlled by master regulators of hypersensitive reaction and pathogenicity (hrp), responsible for transporting avirulent gene (*Avr*). However, *hrp* genes *(hrp*G and *hrp*X) control the transcriptional activity of *Avr* in many bacteria [46, 47]. *Xsa* DD13, *Xal* JG43, and *Xal* FJ1 lacked *hrp* gene cluster and T3SS, while other species had T3SS and *hrp* genes (Fig. S2a). *Xal* JG43 and *Xal* FJ1 had another non-hrp T3SS, Salmonella pathogenicity island-1 (*SPI*-1), which is not essential for xylem colonization and symptoms of sugarcane leaf scald [21]. In contrast, *Xsa* DD13 lacked SPI-1. *Xcc* 8004, *Xoo* PXO99A, and *Xac* 306 had only hrpJ and setJ proteins belonging to the SPI-1 family (Fig. S2b).

### Type IV Secretory System (T4SS)

T4SS plays an essential role in pathogenicity when bacteria deliver DNA or proteins through the secretion system into the host [39]. T4SS was predicted in *Xac* 306, which comprised 12 proteins from VirB1 to VirB11 and VirD4 (Fig. S3a) [48]. VirB5 involved in flagellar movement [49] was unavailable in *Xal* JG43, *Xal* GJ1, *Xsa* DD13, and *Xcc* 8004. VirD4 is deleted in *Xsa* DD13, which is responsible for hydrolyzing ATP to obtain energy to carry out DNA transmission and channel expansion [50, 51]. *Xoo* PXO99A is wholly deprived of T4SS.

### Type V (T5SS) and VI (T6SS) Secretory System

T5SS was present in all studied species (Fig. S3b). Effector proteins (toxins, adhesins, enzymes) are secreted in the Sec-dependent process in T5SS. T5SS transport the proteins with various functions, such as auto-aggregation, invasion, cell-to-cell spread, and cytotoxicity [52–54]. However, T6SS is absent in *Xsa* DD13, *Xal* JG43, *Xal* FJ1, and *Xcc* 8004, whereas *Xac* 306 and *Xoo* PXO99A possessed T6SS (Fig. S3b).

### Potential pathogenic factors

All six strains possessed the cluster genes for the lipopolysaccharide (LPS) transport system, glycogen, two-component system regulators, and three-component system (Fig. S4). The transcription activator-like effectors (TALEs) in *Xanthomonas* deliver effectors by T3SS to induce the expression of host susceptibility (S) genes [55–57]. PthA4 and AvrBs3 of TALEs were present in *Xac* 306 and *Xoo* PXO99A, absent in *Xsa* DD13, *Xal* JG43, *Xal* FJ1, and *Xcc* 8004. However, AvrXa7 existed only in *Xoo* PXO99A.

CRISPR-Cas (CRISPR-associated proteins) is a prokaryotic adaptive immune system that enables rapid and efficient editing of the prokaryotic genome [58]. Except for *Xcc* 8004, genomic features had highlighted that the other five strains had five CRISPR-Cas systems (Fig. S4). Another striking difference among various *Xanthomonas* species is the production of albicidin. *Xal* JG43 and *Xal* FJ1 could produce albicidin but not xanthan due to the missing *gum*M gene. However, no albicidin was produced in *Xac* 306, *Xoo* PXO99A, *Xsa* DD13, and *Xcc* 8004 (Fig. S4).

### Two-component DSF (Diffusible Signal Factor) system

The *rpf* (regulation of pathogenicity factor) gene cluster is involved in cell–cell signaling and control of various cellular processes [59, 60]. The *rpf* cluster was first characterized in *X. campestris pv. campestris*, which comprised nine genes (annotated as *rpfA* through *rpfI*). However, *rpfH* and *rpfI* were missing in *Xal* JG43*, Xal* FJ1, and *Xsa* DD13 (Fig. S6), which are involved in the biosynthesis and detection of diffusible signal factor (DSF). Both *rpfC* (encoding a hybrid two-component DSF sensor) and *rpfG* (encoding a two-component regulator) are implicated in DSF perception and signal transduction [28, 59, 61]. However, *rpfG* is lost in *Xal* FJ1 (Fig. S6).

### Genomic variations associated with virulence of *X. albilineans*

Twenty-three *X. albilineans* strains were recovered from different geographical locations (Table S7) and inoculated in the susceptible sugarcane cultivar (GT46). Their virulence was assayed on the disease index (DI) and Area Under Disease Progress Curve (AUDPC) during the progress of leaf scald in the inoculated plants. The pencil-like lines appeared at 30 DAI (Days after inoculation) in the inoculated plants by 24 strains. Except for NM10, all other strains showed leaf scald symptoms at 90 DAI. Variance analysis indicated that the inoculated strains had significant effects on disease index (*P* ≤ 0.001) and AUDPC (*P* ≤ 0.001) in the fixed model. Twenty-four strains were clustered into three groups based on the disease index and AUDPC during the leaf scald progress of sugarcane. Group #I composed five potent virulent strains, including JG36, JG24, JG43, FS25, and FS63, which displayed the highest disease index and AUDPC. Group# II with mild virulence comprised seven strains, including FS46, JG15, JG37, FS60, FS3, FS35, and FS29, which performed a significantly lower disease index and AUDPC. Group# III consisted of 12 weak virulent strains, including FS5, FS15, FS61, FS53, FS12, NM2, FS62, FS42, FS32, FS7, FS28, and NM10, depicted the least virulence to leaf scald (especially avirulent NM10) (Table 4).

**Table 4:** Disease index(%) and AUDPC of sugarcane leaf scald inoculated by 24 strains of *X. albilineans*

	Potent (Group I)	Mild (Group II)	Weak (Group III)
DAI30	23.5 ± 10.3a	9.2 ± 10.4b	3.0 ± 5.2c
DAI45	36.6 ± 8.0a	20.7 ± 10.0b	10.2 ± 5.0c
DAI60	41.5 ± 10.4a	27.6 ± 5.9b	9.9 ± 5.0c
DAI75	42.5 ± 6.9a	33.2 ± 7.6b	10.1 ± 5.0c
DAI90	53.8 ± 21.3a	38.7 ± 13.1b	10.4 ± 5.4c
AUDPC	2565.1 ± 402.3a	1649.7 ± 303.5b	576.4 ± 296.1c
*Xal* strains	JG43,JG24,JG36, FS63, FS25,	FS46,JG15,JG37,FS60, FS3, FS35, FS29	FS5, FS15, FS61, FS53, FS12, NM2, FS62, FS42, FS32, FS7, FS28, NM10

**Fig. 3:**
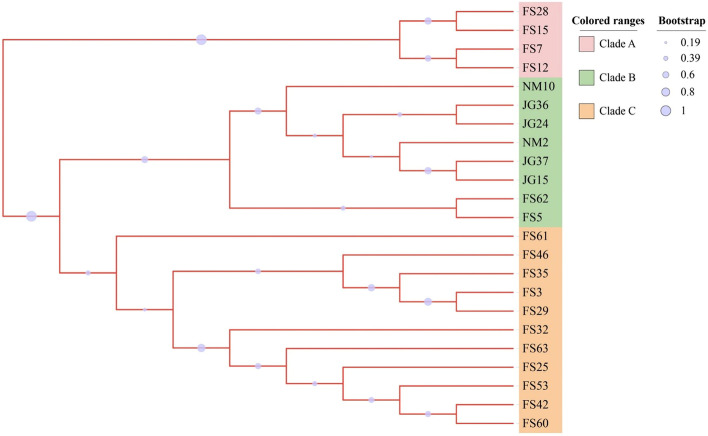
Phylogenetic tree based on SNPs and SV variations with available genotypes in 23 *Xanthomonas* genomes. The phylogenetic tree was constructed using neighbor-joining (NJ) and Bayesian inference (BI). Only branches with ≥ 50% bootstrap support (NJ) and with ≥ 50% posterior probability (BI) are shown in the graph. The numbers on the branches represent the posterior probabilities (%) calculated using Bayesian inference

In order to investigate the genetic basis of virulent diversity, genomes of 23 *Xal* strains were sequenced using Illumina Novaseq 6000. Genomic variations were called using a read-mapping strategy against the *Xal* JG43 as a reference genome, including single-nucleotide polymorphism (SNP) and structural variation (SV). After removing low-quality reads, 3.06 Gb clean reads were generated from 23 *Xal* strains, with a genomic coverage ranging from 257 × to 473 × (Table S7). A total of 69,461 SNPs and 12,523 SVs were obtained from 23 sequenced strains (Table S8). Among these homozygous SNPs, transitions (C: G > T: A and T: A > C: G) were found to be the most common type, whereas transversions (T: A > A: T) were considerably less (Table S9). Out of the structural variations, intrachromosomal rearrangements (ITX) accounted for 83.8%, followed by DEL (7.9%) and INV (6.7%) (Table S8). Genomic variations from 23 sequenced strains were divided into three groups. Clade A with more than 99.5% of SNPs over 2.0 Ti/Tv included FS7, FS12, FS15, and FS28. Clade B with 11,772 SV (94%) composed of eight strains of JG36, JG15, JG24, and JG37 collected from Jinguang, Nanning, and NM2, NM10, FS5, and FS62 from Ningming and Fusui, Chongzuo. The remaining strains with fewer genomic variations in SNPs and SVs were clustered in Clade C (Fig. 3).

**Fig. 4:**
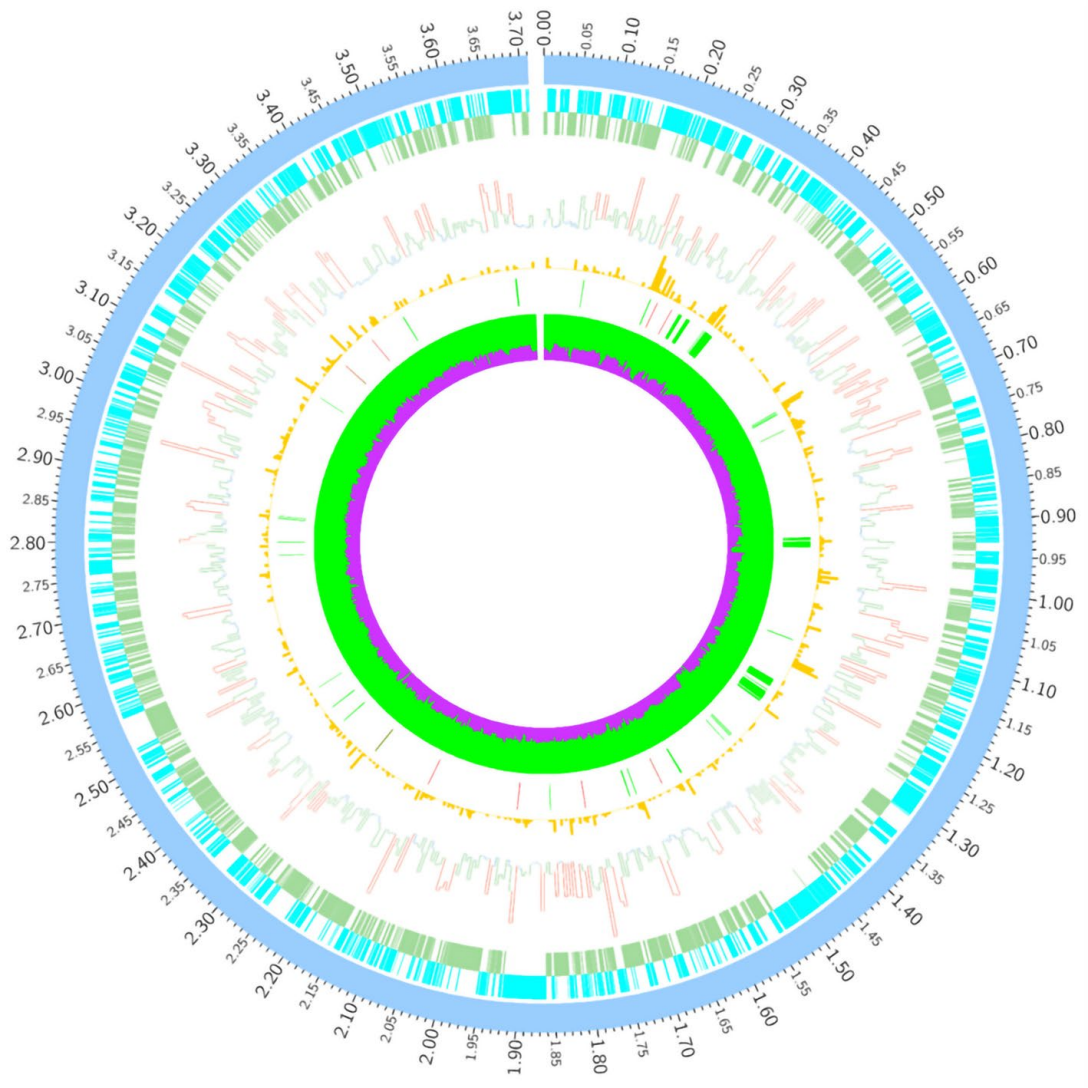
Circular graph depicting the distribution of variants in the 23 sequenced genomes. From outer to inner: the ideogram of the reference *Xal* JG43 genome. The outermost (1^st^) circle was an ideogram of the light blue chromosome, each scale measuring 5 Kb; The 2^nd^ and 3^rd^ circles were genes on the positive and negative chains of the genome; The 4^th^ circle was a histogram of SNP distribution density, red is represented that SNP distribution density more than twice of the genome-wide average level, lower than 1/2 of the genome-wide average level is blue, and the rest is green; The 5^th^ circle was IndeL distribution histogram; The 6^th^ circle was large insertion (INS)and deletion (DEL) distribution, The red part is INS, green represent DEL; The 7^th^ circle was GC content. The green part indicates the GC content in this region; purple represents AT%

**Fig. 5:**
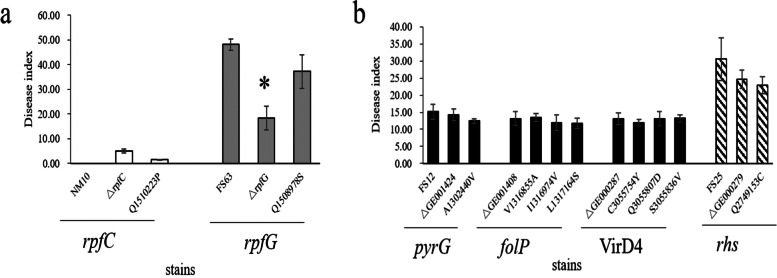
Disease index of NM10, FS12, FS25, FS63, and their single-base mutants.** a** NM10, FS63, and their mutated strains. **b** FS12, FS63, and their mutated strains. Disease indexes were recorded by disease survey 120 days after inoculation. Asterisks (*) indicated the significance at the level of 95%. Three biological replicates were performed for each experiment in this study

A total of 69,461 SNP and 12,523 SV mutations were distributed in the JG43 genome, of which the highest density of InDel appeared in Genomic Island # 5 and # 17. DEL was mainly concentrated in Genomic Islands # 7, # 9, and # 17 (Fig. 4). Genomic Island # 5 contained type IV secretion protein (Rhs and Rhs family protein), whereas Genomic Island # 17 contained type II toxin-antitoxin system of RelE/ParE family toxin. RelE toxins are mRNA interference, while ParE toxins are DNA gyrase (Gyr) inhibitors [62]. Type I toxin-antitoxin system of ptaRNA1 family toxin was involved in Genomic Island #7, which exhibited all the characteristics of a new class of RNA antitoxin by frequent horizontal transfer [63]. Inversion (INS) is a widely distributed structural variation. INS mutations are mainly found in the genes encoding ABC transporter, ATPase component protein, cpsase protein, and modulator of DNA gyrase protein. However, INS mutations were not detected in any pathogenicity gene (Fig. 4, Table S8, Table S10). InDel and DEL are mainly in 20,000 bp-130,000 bp of the JG43 genome, comprising mutations in T1SS, T2SS, and the T3SS family proteins. InDel and DEL concentrate heavily on GI # 17, less involved in pathogenic genes. The *rpf* system and the T6SS are not in the scope of these mutations (Fig. 4, Table S5). Over 1,000 genes with non-synonymous SNPs and 160 with InDel were detected in four weak virulent strains of FS7, FS12, FS15, and FS28 (Table S10). The single-base mutations of SNPs were carried out in FS12, including G/C (1,302,440) in *pyrG* (*GE001424*); T/C (1,316,855), A/G (1,316,974), T/C (1,317,164) in *folP* (*GE001408*); G/A (3,055,754), G/C (3,055,807), T/G (3,055,836) in *VirD4* (*GE000287*). At the same time, C/T SNP at 2,749,153 in the *rhs* family (*GE000279*) was also selected for a single base mutation in the potently virulent strain FS25 (Table 5). The virulence of these FS12 and FS25 mutants was not altered (Table 5; Fig. 5b). However, A/C SNP at 1,510,223 in two-component system *rpfC* gene (*GE001237*) was identified in avirulent NM10. The mutated *rpfC* in NM10 enhanced its pathogenicity with an increased disease index. C/A SNP at 1,508,978 in the two-component *rpfG* gene (*GE001236*) was selected for the potently virulent strain FS63 (Table 5). The virulence of FS63 significantly decreased with a lower disease index after *rpfG* mutation (Fig. 5a), indicating that the *rpf* gene mutation significantly influenced strain virulence.

**Table 5:** SNP mutations detected in the representative sequenced stains

Strains	gene	Position	Codon variation	Amino acid variation	Function
NN10	*GE001237*	1,510,223	cAg/cCg	gln/pro	Two component system rpfC
FS12	*GE001424*	1,302,440	gCg/gTg	ala/val	CTP synthase (pyrG)
FS12	*GE001408*	1,316,855	gTc/gCc	val/ala	dihydropteroate synthase(folP)
FS12	*GE001408*	1,316,974	Att/Gtt	ile/val	dihydropteroate synthase(folP)
FS12	*GE001408*	1,317,164	tTg/tCg	leu/ser	dihydropteroate synthase(folP)
FS12	*GE000287*	3,055,754	tGc/tAc	cys/tyr	type IV secretory system VirD4
FS12	*GE000287*	3,055,807	gaG/gaC	glu/asp	type IV secretory system VirD4
FS12	*GE000287*	3,055,836	Tcc/Gcc	ser/val	type IV secretory system VirD4
FS25	*GE000279*	2,749,153	Cgc/Tgc	arg/cys	type IV secretion protein Rhs
FS63	*GE001236*	1,508,978	Cgt/Agt	arg/ser	Two component system rpfG

## Discussion

Leaf scald disease, caused by *X. albilineans*, is of global interest due to its ability to cause severe economic losses of sugarcane [12]. The results presented here showed that 24 isolates were clustered into three groups: five potent, seven mild virulent, and twelve weak virulent strains of *Xal*. Another weak virulent related strain, *X. sacchari* DD13, has been identified to cause chlorotic streak disease of sugarcane in Guangxi, which showed similar "pencil-like" symptoms as leaf scald at the early stage [14]. The circular genomes of the *X. albilineans* (JG43) and its related strain *Xac* DD13 from China were assembled and annotated using the Oxford Nanopore Technology (ONT) or PacBio RSII technique and corrected by Illumina data for higher consensus accuracy. Compared to *Xac* DD13, *Xal* JG43 reduced its genome by ~ 1.2 Mb and minor repeat sequence. Both JG43 and DD13 did not possess a Hrp-type III secretion system, indicating that the pathogenicity of this pathogen must rely on other virulent factors or secretory systems. However, *Xal*JG43 possesses a T3SS system of the SPI-1 (for Salmonella Pathogenicity Island-1) injectosome family. Compared to DD13, more pathogen-host interaction (PHI) genes and virulent factors were predicted from the database of PHI and VFDB in *Xal* JG43. Bsa T3SS, Rvh T4SS, Trw T4SS, Phytotoxin, and capsular polysaccharides were detected only in JG43. T4SS, one of the critical pathogenic systems, can translocate DNA or proteins into host cells. The evolution of Rvh T4SS was driven by adaptations of the host, indicating that Rvh T4SS can help the pathogen to survive better [31]. Trw T4SS promotes bacteria crossing the inner and outer membrane, which is vital in transporting macromolecules by pathogens [64]. Phytotoxin might destroy the electric potential on the plant membrane, eventually leading to the death of plant cells [34, 65]. The capsular polysaccharide is critical to interfere with cleaning the host phagocytes [66]. These pathogenic factors play an essential role in the infection host, adaptations, and pathogenicity of JG43. The pathogenicity-related system of JG43 was mainly concentrated on genomic islands, whereas the pathogenicity-related genes were involved in prophages in *Xsa* DD13. Blast analysis indicated that 6 of the 17 genomic islands in JG43 were associated with pathogenicity. T4SS is available in GI#1, #3, #7, T3SS in GI # 10, and two-component systems in GI#12 and T2SS in GI#17.

The role of GI in *Xanthomonas* species is diverse and related to bacterial pathogenicity, survival, and evolution [67]. *Xal* JG43 possessed a large number of GI, which reinforced the significance of MGEs in the evolution of the JG43 strain [68]. Detection of 17 GIs in highly potent JG43 strain and 3 GIs in DD13 strain agree with this scenario.

*Xal* JG43 reduced its genome by over 1.0 Mb in size and was absent in T3SS, T6SS, Transcription Activator-Like Effectors (TALEs), and *gum* gene clusters. T3SS is responsible for delivering virulence factors into host cells [69]. Mutant analysis of genes encoding T3SS showed that T3SS is associated with pathogenicity and hypersensitive response (HR) [70]. However, T6SS is involved in various cellular processes such as antibacterial activity, transport of proteins, and biofilm formation [71]. Comparative genomic analysis among *Xanthomonas* species showed that T6SS is absent in *Xal* JG43, *Xal* FJ1, *Xsa* DD13, *Xcc* 8004, while it is available in *Xac* 306 and *Xoo* PXO99A. TALEs are the eukaryotic transcription activator associated with virulence in host species, delivered into host cells through T3SS [72, 73]. The TALEs absence and lack of T3SS in JG43 and DD13 indicated that the pathogenicity of JG43 is not linked with T3SS and T6SS. The absence of TALEs in JG43 and DD13 strains is consistent with previous findings where activation of sugar transporters through TALEs is not vital for inducing virulence [68]. However, instead of TALEs, two TonB-dependent receptors (TBDR) were detected in JG43 and three TBDRs in DD13, which might utilize plant carbohydrates and other biomolecules. These TBDRs are involved in assimilating phenolic compounds and secondary metabolites that assist in disease development by impairing host defense response [74]. *X. albilineans* are exclusively restricted on the xylem. The *gum* cluster is responsible for the production of xanthan and biofilms. In contrast, the absence of xanthan gum allows *X. albilineans* to enter xylem ducts without obstruction and enter the host more smoothly [10, 75]. Genomic comparison of *Xanthomonas* strains revealed that only *X. albilineans* strains (JG43 and FJ1) could produce albicidins in *Xanthomonas spp*, a secreted antibiotic with phytotoxic properties [24]. Albicidin has a tremendous advantage for *X. albilineans* in colonizing xylem against other epiphytes, mainly not possessed by other *Xanthomonas* [75–77]. Albicidin is necessary for *X. albilineans* to invade hosts and inhibit DNA replication in bacteria and sugarcane proplastids [78, 79]. Phytotoxic albicidin is also caused leaf chlorosis and systemic infection by weakening the host immune system [80].

*Xanthomonas* genomes comprise different Mobile Genetic Elements (MGEs), such as transposons (Tn), Insertion Sequence (IS), plasmids, and Genomic Islands (GI), which are associated with virulence factors, genetic variations, and genome structure [67, 81]. Pathogenic factor and SV mutation would cause a significant difference in pathogenicity of JG43 and DD13.

SNP and SV mutation might affect gene loss and structural variation, which leads to gene silencing, overexpression, and interruption of the signal pathway, indicating that SNP and SV mutation might cause a pathogenic difference in the 23 *Xal* strains. Comparative genomic analysis showed that the pathogenicity of our isolated *Xal* strains was associated with their genomic variations. SVs and SNPs among multiple *Xanthomonas* strains suggested that horizontal gene transfer and homologous recombination were associated with their genome evolution [19]. When JG43was used as a reference genome, the highest number of ITX was identified as SVs in highly potent JG24 and JG36 strains. At the same time, SV mutations were mainly concentrated in T1SS, T2SS, and T3SS family proteins. However, the JG24 and JG36 strains remained highly potent, proving that these systems were not crucial for pathogenic systems in *X. albilineans.* Many chromosome translocations affect the genome rearrangement and assembly, leading to genetic diversity and pathogenicity of *Xanthomonas* strains. However, the highest SNP frequency occurred in weak virulent strains, such as FS7, FS12, FS15, and FS28. Critically mutated SNP loci among these strains were mainly distributed in T4SS, GI, and two-component systems. The single-base SNPs mutations in the genes of *pyrG*, *folP,* and *VirD4* of FS12 and *rhs* of FS25 did not alter their virulence. However, the single-base mutation of *rpfC* in avirulent NM10 and *rpfG* in the virulent FS63 altered their pathogenicity, indicating that the *rpf* gene mutation significantly influenced strain virulence. In the quorum-sensing system, *rpfF* and *rpfB* generate DSF [28], which is sensed by the sensor domain of *rpfC* [61]. *RpfC* transmits the signal to *rpfG* for autophosphorylation. After receiving signals, *rpfG* regulates the pathogenic factors by regulating Cyclic-di-GMP concentration through its clp receptor [26, 28]. HY-GDP of *rpfG* combines with two GGDEF domains with the help of the phosphorylated REC domain, forming a complex with *pilZ* to regulate pili movement [28, 82, 83].

### Conclusion

This study sequenced, assembled, and annotated the circular genomes of pathogenic strains; *X. albilineans* (JG43) and *X. sacchari* (DD13). *Xal* JG43 possessed a smaller genome size as compared to *Xsa* DD13. However, JG43 is a highly potent strain as compared to its counterpart. Additionally, we have recovered 23 *Xanthomonas* strains with varying virulence from different sugarcane varieties. These strains were sequenced to obtain SNP mutation sites and other structural variations against the reference genome of JG43. Variation in SNP mutation, virulence factors, and virulence system could lead to differences in pathogenicity. We generated the deletion mutants of *rpfC/rpfG* genes essential for a two-component system. A decreased virulence of mutational △rpfG in FS63 infers that the two-component system is one of the crucial systems in *Xanthomonas* species, associated with pathogenicity and disease progression.

## Materials and methods

### Isolation and identification of *X. albilineans*

Sugarcane leaf samples, displaying pencil-like lines parallel to the veins, were collected from different varieties (Fig. S1). Light-yellow bacterial colonies with smooth, spherical, and shiny surfaces were isolated from leaf samples for purification and culturing using the selective media (SM) described previously [6, 8, 9].

According to the mentioned protocols, the *X. albilineans* strains were determined by PCR with species-specific primers [7, 8] (Table S11). Twenty-five *Xanthonomas* strains were sequenced, including twenty-four from *X. albilineans* and one from *X. sacchari*. Twenty-four sequenced *X. albilineans* strains were further inoculated to the susceptible sugarcane cultivar GT46, the first reported cultivar of sugarcane leaf scald disease, in 2016. Inoculated leaves showed typical symptoms similar to original inoculated bacteria. Blast analysis of the isolated strains confirmed their 100% homology to the initially inoculating strains.

### Inoculation of sugarcane with *X. albilineans*

Sugarcane cultivar (GT46) was inoculated with *X. albilineans* in three independent experiments in a greenhouse. For each strain, ten five-internodes sugarcane plants were inoculated using modified cut-off decapitation [6, 17]. The spindle leaves on a stalk were cut off, and 0.2–0.5 ml of inoculum was then dug onto the cut surface. Inoculum of the inoculated strains was prepared from 2-day-old agar cultures, and bacterial suspensions were calibrated at 1 × 10^6^ CFU/ ml in sterile distilled water. After pretreatment, the bud was soaked in 1 × 10^6^ CFU/ml bacterial solution for five hours, then planted in the pots. The sugarcane single-bud setts were kept in hot water (50 ℃) for five hours for the soaking method. Once symptoms were observed, the infected leaves were collected for bacterial isolation and PCR detection to confirm the inoculated strain.

The disease index (DI) was visually scored at 15-days-interval for each plant after 30 days of inoculation (DAI). The incidence per plant was calculated as the percentage of infected plants out of total inoculated plants. Scores were taken on each plant in five grades of symptom severity [84]: Grade 0, no white pencil stripes; Grade 1, a white pencil-line streak appeared on the leaf; Grade 2, two or more white pencil streaks appear on the leaf; Grade 3, stem and leaf appear yellow or white; Grade 4, plant necrosis or germination of multiple lateral buds; Grade 5, bud or plant death. The rate of virulence for each inoculated strain was calculated as mentioned below [84].

Disease index = ∑[(Number of diseased plants in each grade × value of each grade)/ (Total number of plants investigated × highest grade value)] × 100.

Furthermore, the area under the disease-progress curve (AUDPC) value was calculated [85, 86].

$$AUDPC={\sum }_{i=1}^{n}\left[{(y}_{i+1}+{y}_{i})/2\right]\left({x}_{i+1}-{x}_{i}\right)$$

where AUDPC is the area under the disease progress curve, *y*_*i*_ is the severity of the symptoms at the i^th^ observation; *x*_*i*_—day at the i^th^ observation, and n—the total number of observations.

### Genome sequencing, assembly, and annotation

The genome of *X. albilineans* JG43 was sequenced by Oxford Nanopore Technologies (ONT) and assembled using Canu (V1.5) [87]. Assembled genome was further corrected with Racon (V3.4.3) and Pilon (V1.22), using second-generation data to obtain a high-quality circular genome. However, the genome of *X. sacchari* DD13 was sequenced using SMRT II sequencing technology (Pacific Biosciences, USA), and a complete circular bacterial chromosome was assembled using HGAP software [88]. The genome was cyclized, and the starting sites were adjusted using Circulator (V1.5.5) software. From assembled genomic information, including tRNA, rRNA, repeat sequence, GC content, and gene function, the positional relationship between genomic components was explored using Circos (V0.66) [89].

Assembled genome was analyzed to identify the repeat sequences that were searched against the known repeat sequence database (Repbase) in the bacterial genome using RepeatMasker (V4.0.5) (https://www.repeatmasker.org/) [90]. Non-coding RNAs such as microRNA (miRNA) and small nuclear RNA (snRNA) were predicted using Infernal software (http://eddylab.org/infernal/) [91], while tRNA was annotated by trnascan-SE (http://lowelab.ucsc.edu/tRNAscan-SE/) [92]. The Genewise was used to search for immature stop codons and frameshift mutations in CDS sequences (https://www.ebi.ac.uk/Tools/psa/genewise/) [93]. Genomic islands (GI) in the genome were predicted using Island Path-DiMob (V0.2) (http://www.pathogenomics.sfu.ca/islandviewer/) [94], and prophages were predicted using software PhiSpy (V2.3) (http://phispy.sourceforge.net/) [95].

### Functional annotation of genes

The genes were blasted against the databases of non-redundant proteins (www.ncbi.nlm.nih.gov/refseq/about/nonredundantproteins/) [96], Kyoto Encyclopedia of Genes and Genomes (KEGG) (available at; https://www.genome.jp/kegg/) [97], Swiss-Prot (https://www.expasy.org/resources/uniprotkb-swiss-prot) and TrEMBL (http://www.bioinfo.pte.hu/more/TrEMBL.htm) [98]. Pfam functions were annotated against the Pfam database using HMMER (https://www.ebi.ac.uk/Tools/hmmer/) [99]. Functional annotation of GO and COG against the Nr database was carried out using Blast2GO (https://www.blast2go.com/) [100, 101].

Carbohydrate EnZymes genes were annotated against Carbohydrate Active EnZymes Database (CAZyme) (http://www.cazy.org/) using HMMER software [102]. Transmembrane proteins containing transmembrane helical sugar-based phosphatidylinositol (GPI) and the proteins with a signal peptide were predicted by the software of TMHMM (http://www.cbs.dtu.dk/services/TMHMM/), Kohgpi (http://gpi.unibe.ch/) [103], and SignalP 4.0 (http://www.cbs.dtu.dk/services/SignalP/) [104]. Transmembrane proteins and GPI were removed from proteins containing signal peptides and kept secreted proteins (SP). The resistant genes and their related information were predicted against the Comprehensive Antibiotic Research Database (CARD) (https://card.mcmaster.ca/) using RGI in CARD Database [105]. The virulent genes were predicted by blasting against the Virulence Factor Database of Bacteria (VFDB) (http://www.mgc.ac.cn/VFs/) [106].

### Resequencing and phylogenetic analysis

Single nucleotide polymorphism (SNP) was called against the reference genome of *Xal* JG43 by GATK software (https://gatk.broadinstitute.org/hc/en-us) [107]. The genome of 23 *X. albilineans* strains was sequenced using Illumina Novaseq 6000 platform, with an average coverage of 339 × . The redundant reads (MarkDuplicates) were filtered by Picard software to ensure the detection accuracy of clean reads [108]. A total of 12,523 SNPs were extracted from 23 *X. albilineans*, which were used to construct a phylogenetic tree. After removing ambiguous positions, a final dataset of 17,935 SNPs was generated for each sequence, which was aligned through the neighbor-joining method of MEGA7 software, utilizing the bootstrap value of 1000 replicates [109, 110].

Minimap2 (V2.17) was used to align the 23 sequenced *Xal* genomes with *Xal* JG43, and the aligned reads were sorted using Samtools (V1.12) [109]. BCFtools (V1.12) called single nucleotide variations (including ≤ 50 bp indels) using the haploid model, which was also used to predict the impact of the variations on gene models [110]. BEDTools (V2.29) was used to analyze the presence of homologous segments and the density of SNVs and indels across the reference genome in continuous windows [111]. Circos (V0.69–9) was employed to plot the variation distribution across the reference [89].

### Construction of mutant strain

The single-base mutation was performed by the PNA-directed PCR clamping [112], and two target fragments were fused by PCR, validated by sequencing, and inserted into the pXUK plasmid. The recombinant plasmid was introduced into the corresponding deletions mutants by electric transfer and grown on the plate supplemented with rifampin and kanamycin (Fig. S7).

According to the homologous double exchange construction of the deletion mutant, primers of 500–600 bp DNA upstream and downstream of the target gene were constructed. The enzyme digested the upstream and downstream fragments and connected with the *pK18mob*SacB plasmid to form a recombinant plasmid. The recombinant plasmid was digested, verified, and sequenced. The recombinant plasmid was introduced into the host bacterium by electro-transformation. The upstream and downstream of the target gene underwent single homologous exchange and double homologous exchange with the homologous fragment of the host bacterium. A 10% sucrose was used to screen double homologous exchange. Furthermore, internal and external primers were also used to screen double homologous exchange. Finally, the target gene was deleted (Fig. S5).

## Supplementary Information

**Additional file 1: Fig. S1**. Diseased sugarcane plant with leaf scald disease and chlorotic streak disease symptoms. Left side: *X. albilineans *cause leaf scald disease. (a), (c) and (d) show leaf scald symptoms after *X. albilineans *invade sugarcane; (b) Colony of *X. albilineans *isolated from diseased sugarcane plant; Right side**: ***X. sacchari *cause chlorotic streak disease. (a), (c) and (d) show chlorotic streak symptoms after *X. sacchari *infect sugarcane; (b) Colony of *X. sacchari *isolated from the diseased sugarcane plant. **Fig.** **S2**. Type III secretion system (T3SS) (a), and SPI-1 family (b) of six *Xanthomonas*species. **Fig. S3**. Type IV secretion system (T4SS) (a), T5SS and T6SS (b) of six *Xanthomonas *species. **Fig. S4**. Potential pathogenic factors of six *Xanthomonas *species, including CRISPR system, Lipopolysaccharide transport system protein, Glycogen, Type III secretion regulators, Two-component system regulators, Three-component system, and TALEs. Fig. S5. Verification of *rpfC *and *rpfH *mutations. (a) PCR amplification from the upstream and downstream 500 bp of *rpfC*. M: 2000 bp; Lane 1: *rpfC *Gene left arm; Lane 2: *rpfC *gene right arm. (b) Validation of enzymic fragment ligated with *PK18mob*sacB, a 500bp upstream and downstream fragment of *rpfC *gene. M: 5000 bp; Lane 1,2,3：Validation of *rpfC *recombinant plasmid fragment by enzyme digestion; Lane 4 not included in this experiment. (c) PCR amplified from mutants and its wild type JG43. M:1000 bp; Lane 1, 2, 3; PCR fragment amplified with mutants; Lane 4: PCR fragment amplified with JG43 as template; Lane 5: Water control; Lane 6, 7, 8: Internal primer verification of the target fragment missing in 123, none, which proves the successful deletion of *rpfC *gene; Lang 9: Internal primer fragment of PCR amplified with JG43 as template. (d)PCR validation of *rpfH*gene. M:1000 bp; Lane 1:*Xcc*8004; Lane 2: DD13; Lane 3: JG43; Lane 4: Water control;Lane 5: not included in this experiment. Fig. S6. *rpf *gene cluster of six *Xanthomonas *species. Fig. S7. PCR validation of single-base SNPs mutations. (a) PCR validation of single-base SNP mutations of candidate genes in FS 12. M:2000 bp; Lane 1: 1312440-G-C-L; Lane 2: 1312440-G-C-R; Lane 3: 1316566-A-G-L; Lane 4: 1316566-A-G-R; Lane 5: 1316572-A-G-L; Lane 6: 1316572-A-G-R; Lane 7: 1316840-G-A-L; Lane 8: 1316840-G-A-R; Lane 9: 1316855-T-C-L; Lane10: 1316855-T-C-R; Lane 11: 1316974-A-G-L; lane12: 1316974-A-G-R; lane13: 1317164-T-C-L; Lane 14: 1317164-T-C-R; Lane 15: 3055754-G-A-L; Lane 16: 3055754-G-A-R. **(b) **PCR validation of single-base SNP mutations of candidate genes in FS12 (Lanes 1 and 2), FS25 (Lanes 3 and 4), FS63 (Lanes 5 and 6) and NM10 (Lanes 7 and 8). M:2000 bp; Lane 1: 3055807-G-C-L; Lane 2: 3055807-G-C-R; Lane 3: 2749153-C-T-L; Lane 4: 2749153-C-T-R; Lane 5: 1508978-C-A-L; Lane 6: 1508978-C-A-R; Lane 7:1510223-A-C-L; Lane 8: 1510223-A-C-R. **(c)** SNP point mutation fusion fragment in FS12**. **M:2000 bp; Lane 1: 1312440-G-C; Lane 2: 1316566-A-G; Lane 3: 1316572-A-G; Lane 4:1316840-G-A; Lane 5: 1316855-T-C; Lane 6:1316974-A-G; Lane 7: 1317164-T-C**. (d) **SNP point mutation fusion fragment in FS12. M: 2000 bp; Lane 1:3055754-G-A; Lane 2:3055807-G-C; Lane 3: 3055836-T-G. **(e)** SNP point mutation fusion fragment in FS25 (Lane 1:2749153-C-T), FS63 (Lane 2: 1508978-C-A), and NM10 (Lane 3: 1510223-A-C). M:5000 bp.

**Additional file 2: Table S1**. Basic information of *Xal *JG43 and *Xsa *DD13. **Table S2. **Repeat contents from genome sequence of *Xal *JG43 and *Xsa *DD13. **Table S3**. Genes in plasmid from the genome of *Xal *JG43. (.xls ) **Table S4**. Carbohydrate-active enzymes (CAZys) in *Xal *JG43 and *Xsa *DD13. **Table S5**. Comparative pathogenomics of *X. albilineans *JG43 and its related *X. sacchari *DD13. (.xls ) **Table S6**. Genomic island and prophages of *Xal *JG43 and *Xsa *DD13. (.xls ) **Table S7**. Resequencing of 23 *X. albilineans *strains. **Table S8**. Genomic variations (SNPs and SVs) obtained from 23 sequenced *X. albilineans *strains against JG43. **Table S9**. SNP mutations in 23 strains of *X. albilineans*. **Table S10**. Mutations at the DNA level in 23 strains of *X. albilineans*. **Table S11**. List of primers used in this study.

**Additional file 3.**

## Data Availability

The sequences of resequencing *Xal* genomes have been deposited in the NCBI database (SRR19913200, SRR19913199, SRR19913188, SRR19913184, SRR19913183, SRR19913182, SRR19913181, SRR19913180, SRR19913179, SRR19913178, SRR19913198, SRR19913197, SRR19913196, SRR19913195, SRR19913194, SRR19913193, SRR19913192, SRR19913191, SRR19913190, SRR19913189, SRR19913187, SRR19913186, SRR19913185). The genome assembly and gene annotation have been deposited in the Genome Warehouse (GWH) database with accessions of JAMZGC000000000 (JG43) and CP100647 (DD13).
