# Supplementary figures and images for: Comparative genome analysis unravels pathogenicity of Xanthomonas albilineans causing sugarcane leaf scald disease
Source: BMC Genomics. 2022 Sep 26;23:671. doi: 10.1186/s12864-022-08900-2 (PMC9513982; doi:10.1186/s12864-022-08900-2)

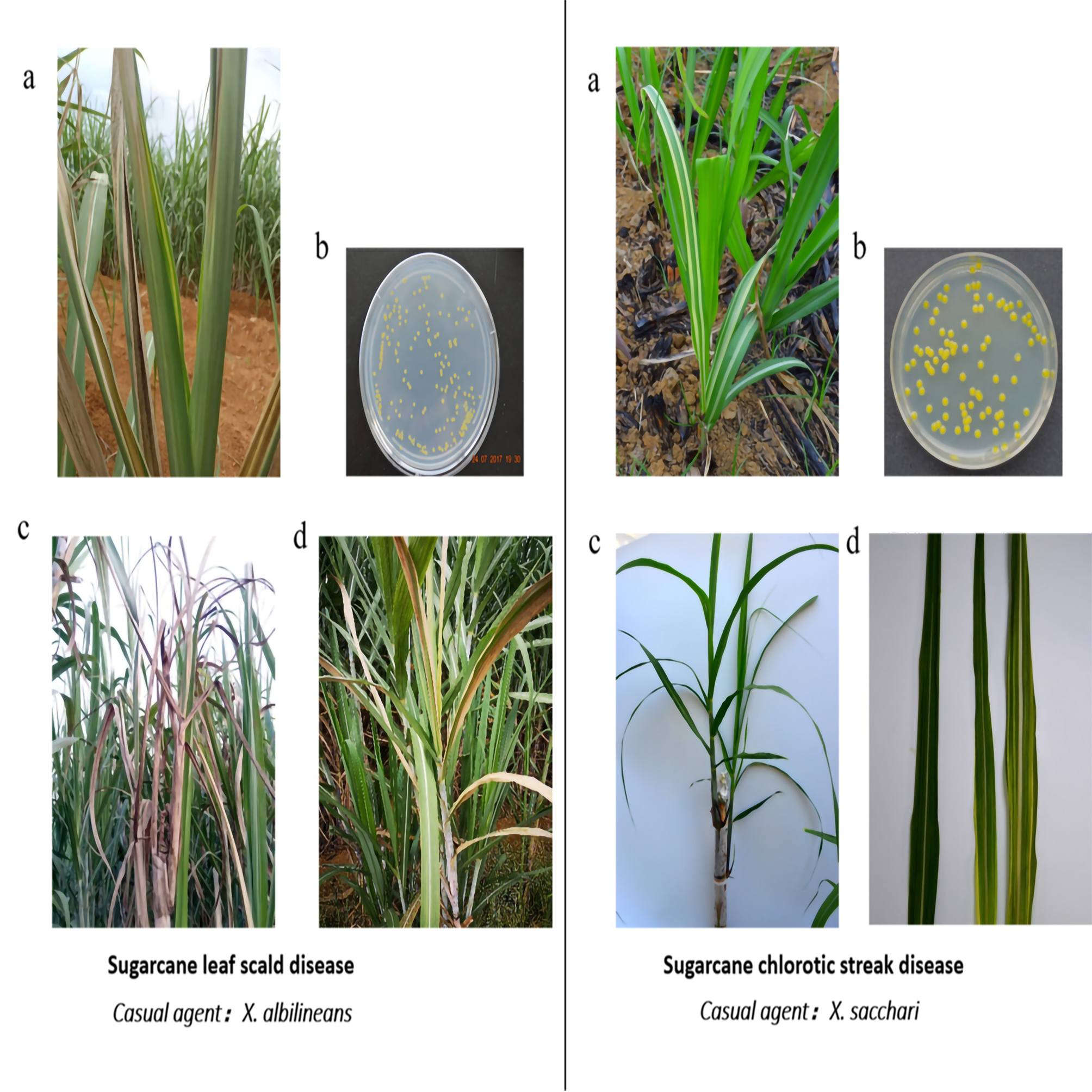

Supplement: Supplementary file 1 — Additional file 1: Fig. S1. Diseased sugarcane plant with leaf scald disease and chlorotic streak disease symptoms. Left side: X. albilineans cause leaf scald disease. (a), (c) and (d) show leaf scald symptoms after X. albilineans invade sugarcane; (b) Colony of X. albilineans isolated from diseased sugarcane plant; Right side: X. sacchari cause chlorotic streak disease. (a), (c) and (d) show chlorotic streak symptoms after X. sacchari infect sugarcane; (b) Colony of X. sacchari isolated from the diseased sugarcane plant. Fig. S2. Type III secretion system (T3SS) (a), and SPI-1 family (b) of six Xanthomonasspecies. Fig. S3. Type IV secretion system (T4SS) (a), T5SS and T6SS (b) of six Xanthomonas species. Fig. S4. Potential pathogenic factors of six Xanthomonas species, including CRISPR system, Lipopolysaccharide transport system protein, Glycogen, Type III secretion regulators, Two-component system regulators, Three-component system, and TALEs. Fig. S5. Verification of rpfC and rpfH mutations. (a) PCR amplification from the upstream and downstream 500 bp of rpfC. M: 2000 bp; Lane 1: rpfC Gene left arm; Lane 2: rpfC gene right arm. (b) Validation of enzymic fragment ligated with PK18mobsacB, a 500bp upstream and downstream fragment of rpfC gene. M: 5000 bp; Lane 1,2,3：Validation of rpfC recombinant plasmid fragment by enzyme digestion; Lane 4 not included in this experiment. (c) PCR amplified from mutants and its wild type JG43. M:1000 bp; Lane 1, 2, 3; PCR fragment amplified with mutants; Lane 4: PCR fragment amplified with JG43 as template; Lane 5: Water control; Lane 6, 7, 8: Internal primer verification of the target fragment missing in 123, none, which proves the successful deletion of rpfC gene; Lang 9: Internal primer fragment of PCR amplified with JG43 as template. (d)PCR validation of rpfHgene. M:1000 bp; Lane 1:Xcc8004; Lane 2: DD13; Lane 3: JG43; Lane 4: Water control;Lane 5: not included in this experiment. Fig. S6. rpf gene cluster of six Xanthomonas sp [file 12864_2022_8900_MOESM1_ESM.zip › Figure S1.tif]

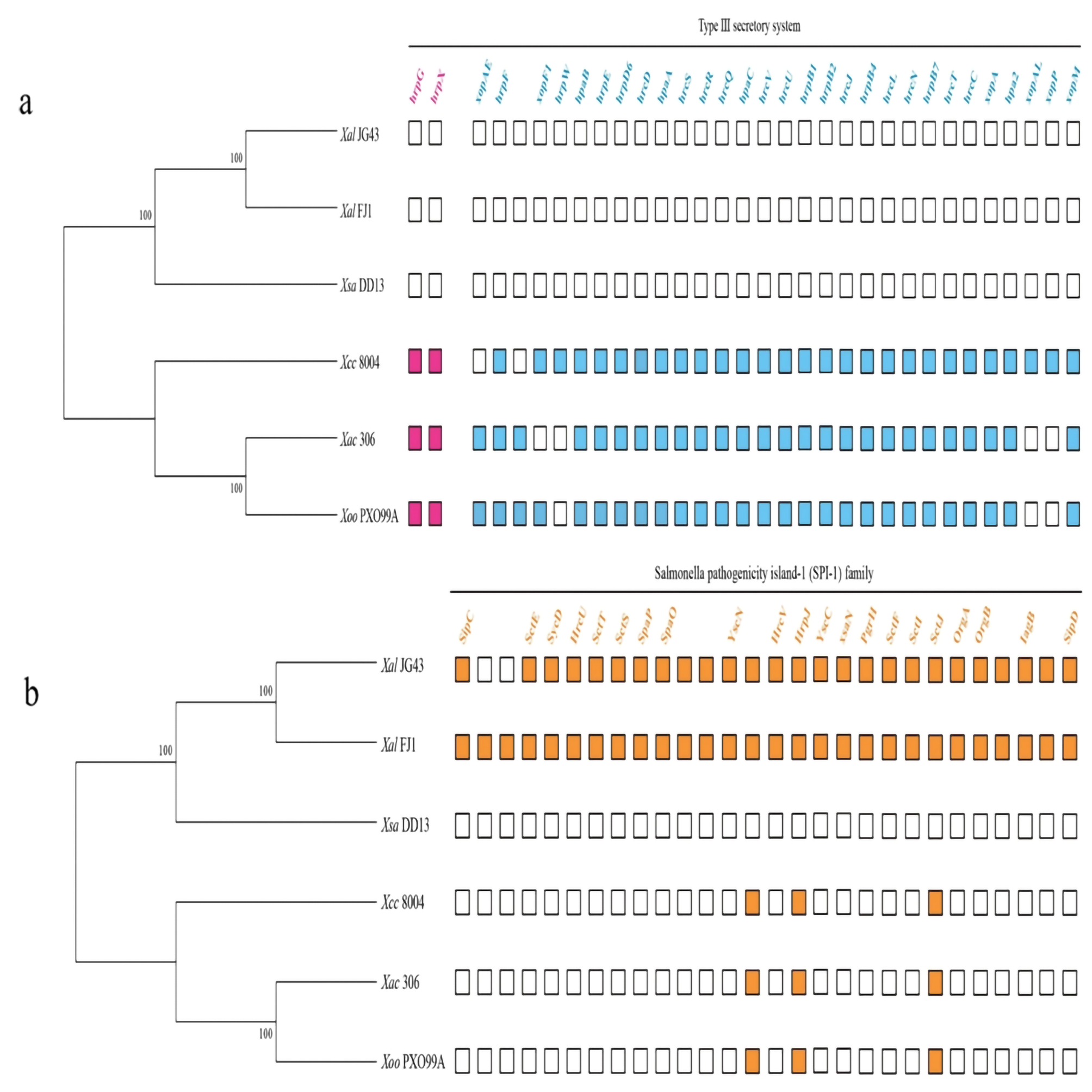

Supplement: Supplementary file 1 — Additional file 1: Fig. S1. Diseased sugarcane plant with leaf scald disease and chlorotic streak disease symptoms. Left side: X. albilineans cause leaf scald disease. (a), (c) and (d) show leaf scald symptoms after X. albilineans invade sugarcane; (b) Colony of X. albilineans isolated from diseased sugarcane plant; Right side: X. sacchari cause chlorotic streak disease. (a), (c) and (d) show chlorotic streak symptoms after X. sacchari infect sugarcane; (b) Colony of X. sacchari isolated from the diseased sugarcane plant. Fig. S2. Type III secretion system (T3SS) (a), and SPI-1 family (b) of six Xanthomonasspecies. Fig. S3. Type IV secretion system (T4SS) (a), T5SS and T6SS (b) of six Xanthomonas species. Fig. S4. Potential pathogenic factors of six Xanthomonas species, including CRISPR system, Lipopolysaccharide transport system protein, Glycogen, Type III secretion regulators, Two-component system regulators, Three-component system, and TALEs. Fig. S5. Verification of rpfC and rpfH mutations. (a) PCR amplification from the upstream and downstream 500 bp of rpfC. M: 2000 bp; Lane 1: rpfC Gene left arm; Lane 2: rpfC gene right arm. (b) Validation of enzymic fragment ligated with PK18mobsacB, a 500bp upstream and downstream fragment of rpfC gene. M: 5000 bp; Lane 1,2,3：Validation of rpfC recombinant plasmid fragment by enzyme digestion; Lane 4 not included in this experiment. (c) PCR amplified from mutants and its wild type JG43. M:1000 bp; Lane 1, 2, 3; PCR fragment amplified with mutants; Lane 4: PCR fragment amplified with JG43 as template; Lane 5: Water control; Lane 6, 7, 8: Internal primer verification of the target fragment missing in 123, none, which proves the successful deletion of rpfC gene; Lang 9: Internal primer fragment of PCR amplified with JG43 as template. (d)PCR validation of rpfHgene. M:1000 bp; Lane 1:Xcc8004; Lane 2: DD13; Lane 3: JG43; Lane 4: Water control;Lane 5: not included in this experiment. Fig. S6. rpf gene cluster of six Xanthomonas sp [file 12864_2022_8900_MOESM1_ESM.zip › Figure S2.tif]

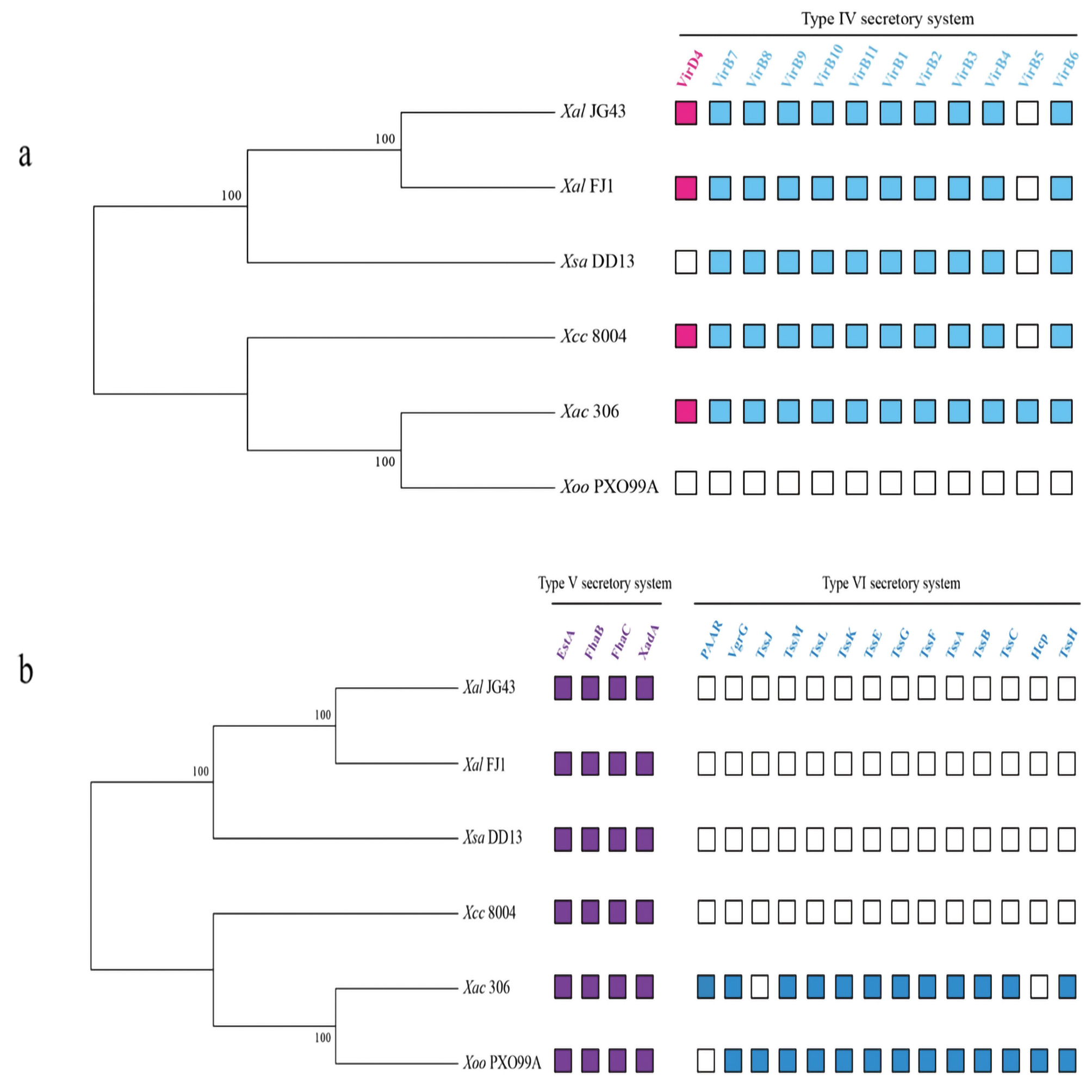

Supplement: Supplementary file 1 — Additional file 1: Fig. S1. Diseased sugarcane plant with leaf scald disease and chlorotic streak disease symptoms. Left side: X. albilineans cause leaf scald disease. (a), (c) and (d) show leaf scald symptoms after X. albilineans invade sugarcane; (b) Colony of X. albilineans isolated from diseased sugarcane plant; Right side: X. sacchari cause chlorotic streak disease. (a), (c) and (d) show chlorotic streak symptoms after X. sacchari infect sugarcane; (b) Colony of X. sacchari isolated from the diseased sugarcane plant. Fig. S2. Type III secretion system (T3SS) (a), and SPI-1 family (b) of six Xanthomonasspecies. Fig. S3. Type IV secretion system (T4SS) (a), T5SS and T6SS (b) of six Xanthomonas species. Fig. S4. Potential pathogenic factors of six Xanthomonas species, including CRISPR system, Lipopolysaccharide transport system protein, Glycogen, Type III secretion regulators, Two-component system regulators, Three-component system, and TALEs. Fig. S5. Verification of rpfC and rpfH mutations. (a) PCR amplification from the upstream and downstream 500 bp of rpfC. M: 2000 bp; Lane 1: rpfC Gene left arm; Lane 2: rpfC gene right arm. (b) Validation of enzymic fragment ligated with PK18mobsacB, a 500bp upstream and downstream fragment of rpfC gene. M: 5000 bp; Lane 1,2,3：Validation of rpfC recombinant plasmid fragment by enzyme digestion; Lane 4 not included in this experiment. (c) PCR amplified from mutants and its wild type JG43. M:1000 bp; Lane 1, 2, 3; PCR fragment amplified with mutants; Lane 4: PCR fragment amplified with JG43 as template; Lane 5: Water control; Lane 6, 7, 8: Internal primer verification of the target fragment missing in 123, none, which proves the successful deletion of rpfC gene; Lang 9: Internal primer fragment of PCR amplified with JG43 as template. (d)PCR validation of rpfHgene. M:1000 bp; Lane 1:Xcc8004; Lane 2: DD13; Lane 3: JG43; Lane 4: Water control;Lane 5: not included in this experiment. Fig. S6. rpf gene cluster of six Xanthomonas sp [file 12864_2022_8900_MOESM1_ESM.zip › Figure S3.tif]

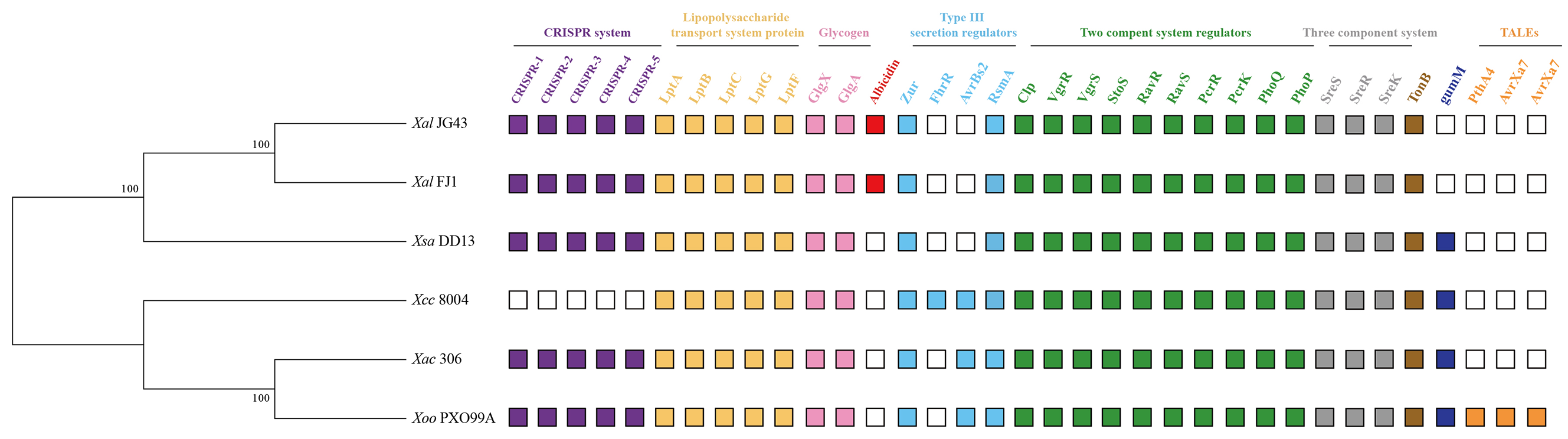

Supplement: Supplementary file 1 — Additional file 1: Fig. S1. Diseased sugarcane plant with leaf scald disease and chlorotic streak disease symptoms. Left side: X. albilineans cause leaf scald disease. (a), (c) and (d) show leaf scald symptoms after X. albilineans invade sugarcane; (b) Colony of X. albilineans isolated from diseased sugarcane plant; Right side: X. sacchari cause chlorotic streak disease. (a), (c) and (d) show chlorotic streak symptoms after X. sacchari infect sugarcane; (b) Colony of X. sacchari isolated from the diseased sugarcane plant. Fig. S2. Type III secretion system (T3SS) (a), and SPI-1 family (b) of six Xanthomonasspecies. Fig. S3. Type IV secretion system (T4SS) (a), T5SS and T6SS (b) of six Xanthomonas species. Fig. S4. Potential pathogenic factors of six Xanthomonas species, including CRISPR system, Lipopolysaccharide transport system protein, Glycogen, Type III secretion regulators, Two-component system regulators, Three-component system, and TALEs. Fig. S5. Verification of rpfC and rpfH mutations. (a) PCR amplification from the upstream and downstream 500 bp of rpfC. M: 2000 bp; Lane 1: rpfC Gene left arm; Lane 2: rpfC gene right arm. (b) Validation of enzymic fragment ligated with PK18mobsacB, a 500bp upstream and downstream fragment of rpfC gene. M: 5000 bp; Lane 1,2,3：Validation of rpfC recombinant plasmid fragment by enzyme digestion; Lane 4 not included in this experiment. (c) PCR amplified from mutants and its wild type JG43. M:1000 bp; Lane 1, 2, 3; PCR fragment amplified with mutants; Lane 4: PCR fragment amplified with JG43 as template; Lane 5: Water control; Lane 6, 7, 8: Internal primer verification of the target fragment missing in 123, none, which proves the successful deletion of rpfC gene; Lang 9: Internal primer fragment of PCR amplified with JG43 as template. (d)PCR validation of rpfHgene. M:1000 bp; Lane 1:Xcc8004; Lane 2: DD13; Lane 3: JG43; Lane 4: Water control;Lane 5: not included in this experiment. Fig. S6. rpf gene cluster of six Xanthomonas sp [file 12864_2022_8900_MOESM1_ESM.zip › Figure S4.tif]

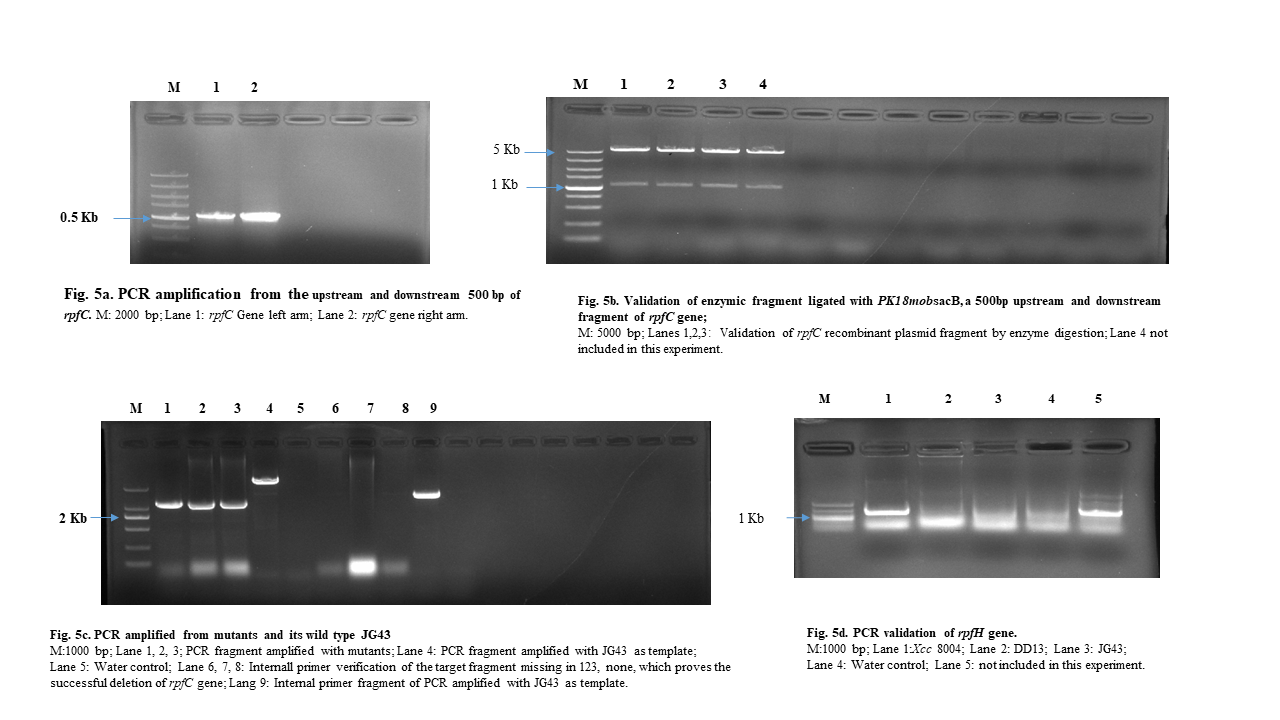

Supplement: Supplementary file 1 — Additional file 1: Fig. S1. Diseased sugarcane plant with leaf scald disease and chlorotic streak disease symptoms. Left side: X. albilineans cause leaf scald disease. (a), (c) and (d) show leaf scald symptoms after X. albilineans invade sugarcane; (b) Colony of X. albilineans isolated from diseased sugarcane plant; Right side: X. sacchari cause chlorotic streak disease. (a), (c) and (d) show chlorotic streak symptoms after X. sacchari infect sugarcane; (b) Colony of X. sacchari isolated from the diseased sugarcane plant. Fig. S2. Type III secretion system (T3SS) (a), and SPI-1 family (b) of six Xanthomonasspecies. Fig. S3. Type IV secretion system (T4SS) (a), T5SS and T6SS (b) of six Xanthomonas species. Fig. S4. Potential pathogenic factors of six Xanthomonas species, including CRISPR system, Lipopolysaccharide transport system protein, Glycogen, Type III secretion regulators, Two-component system regulators, Three-component system, and TALEs. Fig. S5. Verification of rpfC and rpfH mutations. (a) PCR amplification from the upstream and downstream 500 bp of rpfC. M: 2000 bp; Lane 1: rpfC Gene left arm; Lane 2: rpfC gene right arm. (b) Validation of enzymic fragment ligated with PK18mobsacB, a 500bp upstream and downstream fragment of rpfC gene. M: 5000 bp; Lane 1,2,3：Validation of rpfC recombinant plasmid fragment by enzyme digestion; Lane 4 not included in this experiment. (c) PCR amplified from mutants and its wild type JG43. M:1000 bp; Lane 1, 2, 3; PCR fragment amplified with mutants; Lane 4: PCR fragment amplified with JG43 as template; Lane 5: Water control; Lane 6, 7, 8: Internal primer verification of the target fragment missing in 123, none, which proves the successful deletion of rpfC gene; Lang 9: Internal primer fragment of PCR amplified with JG43 as template. (d)PCR validation of rpfHgene. M:1000 bp; Lane 1:Xcc8004; Lane 2: DD13; Lane 3: JG43; Lane 4: Water control;Lane 5: not included in this experiment. Fig. S6. rpf gene cluster of six Xanthomonas sp [file 12864_2022_8900_MOESM1_ESM.zip › Figure S5.tif]

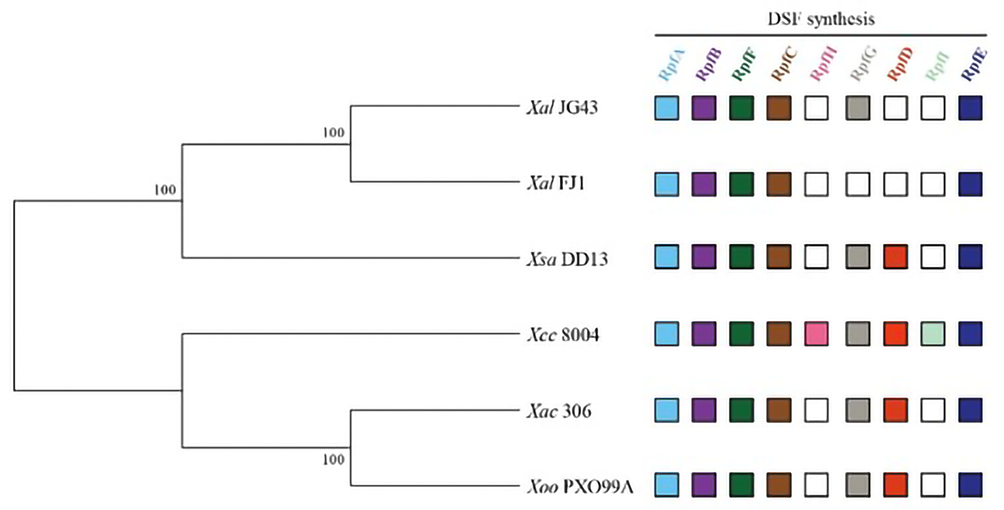

Supplement: Supplementary file 1 — Additional file 1: Fig. S1. Diseased sugarcane plant with leaf scald disease and chlorotic streak disease symptoms. Left side: X. albilineans cause leaf scald disease. (a), (c) and (d) show leaf scald symptoms after X. albilineans invade sugarcane; (b) Colony of X. albilineans isolated from diseased sugarcane plant; Right side: X. sacchari cause chlorotic streak disease. (a), (c) and (d) show chlorotic streak symptoms after X. sacchari infect sugarcane; (b) Colony of X. sacchari isolated from the diseased sugarcane plant. Fig. S2. Type III secretion system (T3SS) (a), and SPI-1 family (b) of six Xanthomonasspecies. Fig. S3. Type IV secretion system (T4SS) (a), T5SS and T6SS (b) of six Xanthomonas species. Fig. S4. Potential pathogenic factors of six Xanthomonas species, including CRISPR system, Lipopolysaccharide transport system protein, Glycogen, Type III secretion regulators, Two-component system regulators, Three-component system, and TALEs. Fig. S5. Verification of rpfC and rpfH mutations. (a) PCR amplification from the upstream and downstream 500 bp of rpfC. M: 2000 bp; Lane 1: rpfC Gene left arm; Lane 2: rpfC gene right arm. (b) Validation of enzymic fragment ligated with PK18mobsacB, a 500bp upstream and downstream fragment of rpfC gene. M: 5000 bp; Lane 1,2,3：Validation of rpfC recombinant plasmid fragment by enzyme digestion; Lane 4 not included in this experiment. (c) PCR amplified from mutants and its wild type JG43. M:1000 bp; Lane 1, 2, 3; PCR fragment amplified with mutants; Lane 4: PCR fragment amplified with JG43 as template; Lane 5: Water control; Lane 6, 7, 8: Internal primer verification of the target fragment missing in 123, none, which proves the successful deletion of rpfC gene; Lang 9: Internal primer fragment of PCR amplified with JG43 as template. (d)PCR validation of rpfHgene. M:1000 bp; Lane 1:Xcc8004; Lane 2: DD13; Lane 3: JG43; Lane 4: Water control;Lane 5: not included in this experiment. Fig. S6. rpf gene cluster of six Xanthomonas sp [file 12864_2022_8900_MOESM1_ESM.zip › Figure S6.tif]

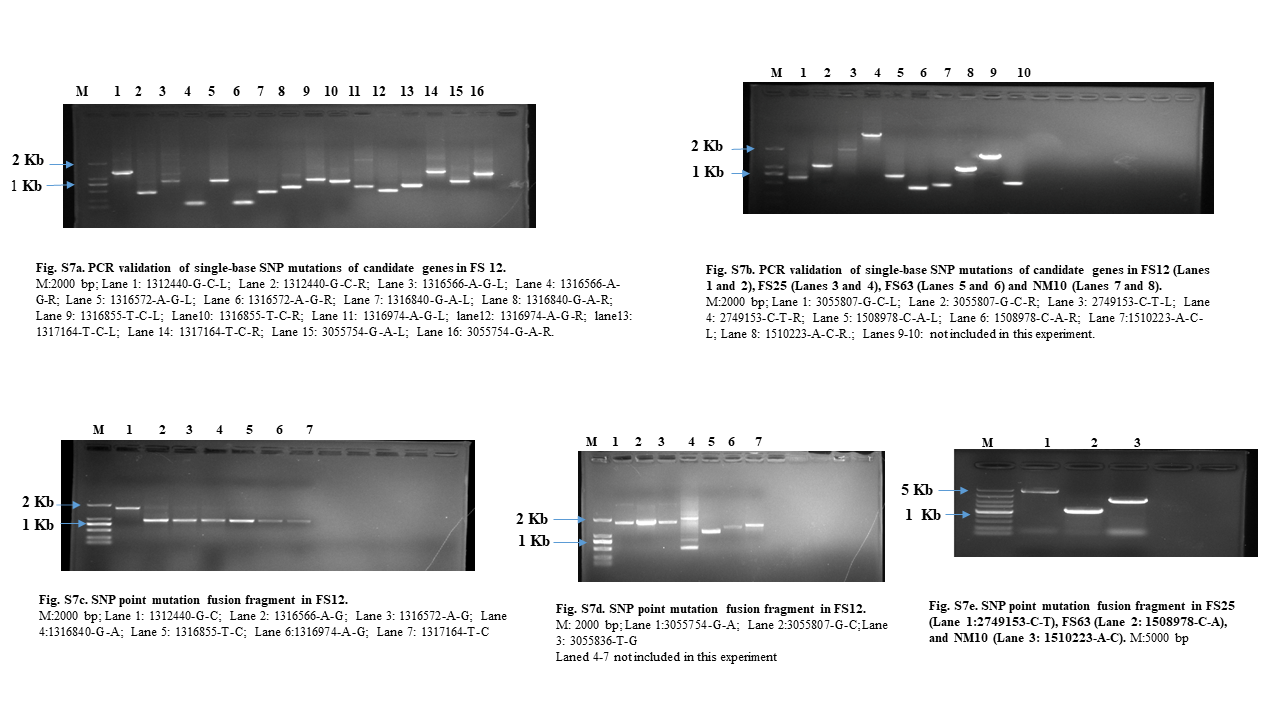

Supplement: Supplementary file 1 — Additional file 1: Fig. S1. Diseased sugarcane plant with leaf scald disease and chlorotic streak disease symptoms. Left side: X. albilineans cause leaf scald disease. (a), (c) and (d) show leaf scald symptoms after X. albilineans invade sugarcane; (b) Colony of X. albilineans isolated from diseased sugarcane plant; Right side: X. sacchari cause chlorotic streak disease. (a), (c) and (d) show chlorotic streak symptoms after X. sacchari infect sugarcane; (b) Colony of X. sacchari isolated from the diseased sugarcane plant. Fig. S2. Type III secretion system (T3SS) (a), and SPI-1 family (b) of six Xanthomonasspecies. Fig. S3. Type IV secretion system (T4SS) (a), T5SS and T6SS (b) of six Xanthomonas species. Fig. S4. Potential pathogenic factors of six Xanthomonas species, including CRISPR system, Lipopolysaccharide transport system protein, Glycogen, Type III secretion regulators, Two-component system regulators, Three-component system, and TALEs. Fig. S5. Verification of rpfC and rpfH mutations. (a) PCR amplification from the upstream and downstream 500 bp of rpfC. M: 2000 bp; Lane 1: rpfC Gene left arm; Lane 2: rpfC gene right arm. (b) Validation of enzymic fragment ligated with PK18mobsacB, a 500bp upstream and downstream fragment of rpfC gene. M: 5000 bp; Lane 1,2,3：Validation of rpfC recombinant plasmid fragment by enzyme digestion; Lane 4 not included in this experiment. (c) PCR amplified from mutants and its wild type JG43. M:1000 bp; Lane 1, 2, 3; PCR fragment amplified with mutants; Lane 4: PCR fragment amplified with JG43 as template; Lane 5: Water control; Lane 6, 7, 8: Internal primer verification of the target fragment missing in 123, none, which proves the successful deletion of rpfC gene; Lang 9: Internal primer fragment of PCR amplified with JG43 as template. (d)PCR validation of rpfHgene. M:1000 bp; Lane 1:Xcc8004; Lane 2: DD13; Lane 3: JG43; Lane 4: Water control;Lane 5: not included in this experiment. Fig. S6. rpf gene cluster of six Xanthomonas sp [file 12864_2022_8900_MOESM1_ESM.zip › Figure S7.tif]
