# Supplementary material for: Comparative genome analysis unravels pathogenicity of Xanthomonas albilineans causing sugarcane leaf scald disease
Source: BMC Genomics. 2022 Sep 26;23:671. doi: 10.1186/s12864-022-08900-2 (PMC9513982; doi:10.1186/s12864-022-08900-2)
Supplement: Supplementary file 2 — Additional file 2: Table S1. Basic information of Xal JG43 and Xsa DD13. Table S2. Repeat contents from genome sequence of Xal JG43 and Xsa DD13. Table S3. Genes in plasmid from the genome of Xal JG43. (.xls ) Table S4. Carbohydrate-active enzymes (CAZys) in Xal JG43 and Xsa DD13. Table S5. Comparative pathogenomics of X. albilineans JG43 and its related X. sacchari DD13. (.xls ) Table S6. Genomic island and prophages of Xal JG43 and Xsa DD13. (.xls ) Table S7. Resequencing of 23 X. albilineans strains. Table S8. Genomic variations (SNPs and SVs) obtained from 23 sequenced X. albilineans strains against JG43. Table S9. SNP mutations in 23 strains of X. albilineans. Table S10. Mutations at the DNA level in 23 strains of X. albilineans. Table S11. List of primers used in this study. [file 12864_2022_8900_MOESM2_ESM.pdf]

Supplementary Information

Table S1. Basic information of *Xal* JG43 and *Xsa* DD13.

Table S2. Repeat contents from genome sequence of *Xal* JG43 and *Xsa* DD13.

Table S3. Genes in plasmid from the genome of *Xal* JG43. (.xls)

Table S4. Carbohydrate-active enzymes (CAZys) in *Xal* JG43 and *Xsa* DD13.

Table S5. Comparative pathogenomics of *X. albilineans* JG43 and its related *X. sacchari* DD13. (.xls)

Table S6. Genomic island and prophages of *Xal* JG43 and *Xsa* DD13. (.xls)

Table S7. Resequencing of 23 *X. albilineans* strains.

Table S8. Genomic variations (SNPs and SVs) obtained from 23 sequenced *X. albilineans* strains against JG43.

Table S9. SNP mutations in 23 strains of *X. albilineans*.

Table S10. Mutations at the DNA level in 23 strains of *X. albilineans*.

Table S11. List of primers used in this study.

Fig. S1. Diseased sugarcane plant with leaf scald disease and chlorotic streak disease symptoms.

Left side: *X. albilineans* cause leaf scald disease. (a), (c) and (d) show leaf scald symptoms after *X. albilineans* invade sugarcane; (b) Colony of *X. albilineans* isolated from diseased sugarcane plant;

Right side: *X. sacchari* cause chlorotic streak disease. (a), (c) and (d) show chlorotic streak symptoms after *X. sacchari* infect sugarcane; (b) Colony of *X. sacchari* isolated from the diseased sugarcane plant.

Fig. S2. Type III secretion system (T3SS) (a), and SPI-1 family (b) of six *Xanthomonas* species.

Fig. S3. Type IV secretion system (T4SS) (a), T5SS and T6SS (b) of six *Xanthomonas* species.

Fig. S4. Potential pathogenic factors of six *Xanthomonas* species, including CRISPR system, Lipopolysaccharide transport system protein, Glycogen, Type III secretion

regulators, Two-component system regulators, Three-component system, and TALEs.

Fig. S5. Verification of *rpfC* and *rpfH* mutations. (a) PCR amplification from the upstream and downstream 500 bp of *rpfC*. M: 2000 bp; Lane 1: *rpfC* Gene left arm; Lane 2: *rpfC* gene right arm. **(b) Validation of enzymic fragment ligated with *PK18mob*sacB, a 500bp upstream and downstream fragment of *rpfC* gene**. M: 5000 bp; Lane 1,2,3: Validation of *rpfC* recombinant plasmid fragment by enzyme digestion; Lane 4 not included in this experiment. (**c) PCR amplified from mutants and its wild type JG43.** M:1000 bp; Lane 1, 2, 3; PCR fragment amplified with mutants; Lane 4: PCR fragment amplified with JG43 as template; Lane 5: Water control; Lane 6, 7, 8: Internal primer verification of the target fragment missing in 123, none, which proves the successful deletion of *rpfC* gene; Lang 9: Internal primer fragment of PCR amplified with JG43 as template. **(d) PCR validation of *rpfH* gene.** M:1000 bp; Lane 1: *Xcc* 8004; Lane 2: DD13; Lane 3: JG43; Lane 4: Water control; Lane 5: not included in this experiment.

Fig. S6. *rpf* gene cluster of six *Xanthomonas* species.

Fig. S7. PCR validation of single-base SNPs mutations. (a) PCR validation of single-base SNP mutations of candidate genes in FS 12. M:2000 bp; Lane 1: 1312440-G-C-L; Lane 2: 1312440-G-C-R; Lane 3: 1316566-A-G-L; Lane 4: 1316566-A-G-R; Lane 5: 1316572-A-G-L; Lane 6: 1316572-A-G-R; Lane 7: 1316840-G-A-L; Lane 8: 1316840-G-A-R; Lane 9: 1316855-T-C-L; Lane10: 1316855-T-C-R; Lane 11: 1316974-A-G-L; lane12: 1316974-A-G-R; lane13: 1317164-T-C-L; Lane 14: 1317164-T-C-R; Lane 15: 3055754-G-A-L; Lane 16: 3055754-G-A-R. **(b) PCR validation of single-base SNP mutations of candidate genes in FS12 (Lanes 1 and 2), FS25 (Lanes 3 and 4), FS63 (Lanes 5 and 6) and NM10 (Lanes 7 and 8).** M:2000 bp; Lane 1: 3055807-G-C-L; Lane 2: 3055807-G-C-R; Lane 3: 2749153-C-T-L; Lane 4: 2749153-C-T-R; Lane 5: 1508978-C-A-L; Lane 6: 1508978-C-A-R; Lane 7:1510223-A-C-L; Lane 8: 1510223-A-C-R. **(c) SNP point mutation fusion fragment in FS12.** M:2000 bp; Lane 1: 1312440-G-C; Lane 2: 1316566-A-G; Lane 3: 1316572-A-G; Lane 4:1316840-G-A; Lane 5: 1316855-T-C; Lane 6:1316974-A-G; Lane 7: 1317164-T-C**. (d) SNP point mutation fusion fragment in FS12.** M: 2000 bp; Lane 1:3055754-G-A; Lane 2:3055807-G-C; Lane 3: 3055836-T-G**. (e) SNP point mutation fusion fragment in FS25 (Lane 1:2749153-C-T), FS63 (Lane 2: 1508978-C-A), and NM10 (Lane 3: 1510223-A-C).** M:5000 bp.
