# Supplementary material for: Comparative genome analysis unravels pathogenicity of Xanthomonas albilineans causing sugarcane leaf scald disease
Source: BMC Genomics. 2022 Sep 26;23:671. doi: 10.1186/s12864-022-08900-2 (PMC9513982; doi:10.1186/s12864-022-08900-2)
Supplement: Supplementary file 3 — Additional file 3. [file 12864_2022_8900_MOESM3_ESM.zip › Table S10.docx]

**Table S10. Mutations at DNA level in 23 strains of *Xanthomonas* species.**

| **Strain** | **Genes with Non-synonymous SNP** | **Genes with InDel** | **Genes with SV** |
| --- | --- | --- | --- |
| JG15 | 3 | 0 | 38 |
| JG24 | 6 | 0 | 2 |
| JG36 | 4 | 0 | 2 |
| JG37 | 4 | 0 | 35 |
| NM10 | 7 | 0 | 56 |
| NM2 | 4 | 0 | 14 |
| FS3 | 4 | 2 | 96 |
| FS5 | 5 | 4 | 173 |
| FS7 | 1108 | 162 | 74 |
| FS12 | 1110 | 162 | 97 |
| FS15 | 1110 | 161 | 112 |
| FS25 | 5 | 3 | 70 |
| FS28 | 1112 | 164 | 84 |
| FS29 | 3 | 3 | 88 |
| FS32 | 5 | 3 | 99 |
| FS35 | 4 | 2 | 90 |
| FS42 | 5 | 3 | 71 |
| FS46 | 6 | 1 | 36 |
| FS53 | 4 | 1 | 147 |
| FS60 | 5 | 2 | 60 |
| FS61 | 8 | 2 | 56 |
| FS62 | 6 | 1 | 69 |
| FS63 | 5 | 2 | 94 |

Note: InDel: Insertion and Deletion.
