# Supplementary material for: Comparative genome analysis unravels pathogenicity of Xanthomonas albilineans causing sugarcane leaf scald disease
Source: BMC Genomics. 2022 Sep 26;23:671. doi: 10.1186/s12864-022-08900-2 (PMC9513982; doi:10.1186/s12864-022-08900-2)
Supplement: Supplementary file 3 — Additional file 3. [file 12864_2022_8900_MOESM3_ESM.zip › Table S11.docx]

**Table S11. The sequences of primers used in the analysis.**

|  | **Primer pairs** | **Target sequence (5'-3')** | **Species-specificity** | **References** |
| --- | --- | --- | --- | --- |
| P1 | Xar  xaf | CGATCAGCGATGCACGCAGT | ABC transportprotein | 10 |
|  |  | CCTGGTGATGACGCTGGGTT | ABC transportprotein |  |
| P2 | atpD-F  atpD-R2 | ATGGCCAACGAACGTCCTGC | atpD | 10 |
|  |  | AACTTGTAGCCGCGACGGTATTC |  |  |
| P3 | XgyrB1F  XgyrB1R2 | CCCATCAAGGTGCTGAAAAT | *gyrB* | 10,11 |
|  |  | GGGCGGWGTGTACAAGGC |  |  |
| P4 | glnA-F2  glnA-R2 | GGTTAAGGACAACAAGATCG | GlnA | 10 |
|  |  | GCGGCRAAGGTCAGGTAC | GlnA |  |
| P5 | rpoD-F  rpoD-R3 | ATGGCCAACGAACGTCCTGC | RpoD | 10,11 |
|  |  | AACTTGTAGCCGCGACGGTATTC | RpoD |  |
| P6 | virB6-12F  virB6-12R | CTGTTCACCGGTCAGAAGGA | VirB6 | 10 |
|  |  | CTCGCCGTCATCTTCAATGC | VirB6 |  |
| P7 | virB6-13F  virB6-13R | CATTGAAGATGACGGCGAG | VirB6 | In this study |
|  |  | CTCGCGCGAGGCATTAGAAG | VirB6 |  |
| P8 | virB6-22F | ACGAGTACAACCCGGACAA | VirB6 | In this study |
|  |  | CCCATCAAGGTGCTGAAAAT | VirB6 |  |
| P9 | virB6-23F | ATGCGGAAGGCTTGTCCTCG | VirB6 | In this study |
|  |  | CGTGTAAGGCGCTCGAATGA | VirB6 |  |
| P14 | rpfG-L-F | CCCGGATCC TGTTCATCAATGGCAAGGAAAT | Mutant of rpfG | In this study |
|  | rpfG-L-R | CCCTCTAGA ATTATCCATCTCCGCCGTCATC |  |  |
| P15 | rpfG-R-F | CCCTCTAGA TCGCGAGCAACTGCAGGTGGTCT |  |  |
|  | rpfG-R-R | CCCAAGCTT AATAAGGCGTCACGAGGATCGT |  |  |
| P16 | ny-rpfG-L | GAATTGCGCGCACGTTGTCACA | Inner primer of rpfG | In this study |
|  | ny-rpfG-R | AATCGCTTCGCCGACCAATCCA |  |  |
| P18 | GE001424-G-C-F | GATGTTgcgGTGGAGATCGGTGGCACCGTCGG | Full-length primer of GM001424 | In this study |
|  | GE001424- G-C-R | ATCTCCACcgcAACATCGAAGCCGGCGGTGGC |  |  |
| P19 | GE001424-D-F | CCCTCTAGA ATGACCCCCCTGATTTTCGTGACT | Primer of 1302440,G-C | In this study |
|  | GE001424-D-R | CCCAAGCTT TCAGGCACGCGCTTCCTTCAAC |  |  |
| P20 | GE001408-A-G-F | CGAGCGTattTTCGCTGCGGAGATGGCCGGCA | Primer of 1316974,A-G | In this study |
|  | GE001408-A-G-R | CAGCGAAaatACGCTCGACCAGAAATCGATGC |  |  |
| P21 | GE001408-T-C-F | ACGCGAGttgCCGGAACAGCGCGTGGCCGGTT | Primer of 11317164,T-C | In this study |
|  | GE001408-T-C-R | GTTCCGGcaaCTCGCGTCCGGTCAACTCGCCG |  |  |
| P22 | GE000287-D-F | CCCTCTAGA ATGCGATTACGCTCCGCCCAGCG | Primer of GE000287 | In this study |
|  | GE000287-D-R | CCCAAGCTT CTACCCGTTGCCATCGCTGGGC |  |  |
| P23 | GE000287-G-A-F | AAAGGtcttgcGGCCCATTGCCGGCCAGAACGG | Primer of 3055754,T-C | In this study |
|  | GE000287-G-A-R | ATGGGCCgcaagaCCTTTGTGGCTGCCTGCGAA |  |  |
| P24 | GE000287-G-C-F | TACAGCGTGgaggcgATGGCGACGATGTCGCCG | Primer of 3055807,T-C | In this study |
|  | GE000287-G-C-R | CATcgcctcCACGCTGTACAAGGAGATGAGGGG |  |  |
| P25 | GE000287-T-G-F | ATTCCAAcgatccGTGCTGCCGTCATCACTGAAC | Primer of 13055836,T-C | In this study |
|  | GE000287-T-G-R | AGCACggatcgTTGGAATCCACTGGGCTACGTG |  |  |
| P26 | GE001408-T-C-F | ACGCGAGttgCCGGAACAGCGCGTGGCCGGTT | Primer of 1317164,T-C | In this study |
|  | GE001408-T-C-R | GTTCCGGcaaCTCGCGTCCGGTCAACTCGCCG |  |  |
| P27 | GE001408-D-F | CCCTCTAGA ATGTTCGATACCTCGCCTCGCCTGG | Full-length primer of GE001408 | In this study |
|  | GE001408-D-R | CCCAAGCTT TCAGTCCTCGTCCGGCCAGCGG |  |  |
| P28 | GE000279-C-G-F | AAACCTACcgcTACGACGGCAACGGCCGCCGC | Primer of 2749153,C-T | In this study |
|  | GE000279-C-G-R | GTCGTAgcgGTAGGTTTCCTTACCGGTCGCTT |  |  |
| P29 | GE000279-D-F | CCCTCTAGA ATGGATAGGTTCCAGGCGTTTT | Full-length primer of GE000287 | In this study |
|  | GE0002279-D-R | CCCAAGCTT TCAACTATTCAGTAGCAATTGA |  |  |
| P30 | GE001236-C-A-F | AACCAAGGAGGCAcagCGCGCCAACGAAGCCAAG | Primer of 1508978,A-C | In this study |
|  | GE001236-C-A-R | GctgTGCCTCCTTGGTTGCCCGAGTCAACTGC |  |  |
| P31 | GE001236-D-F | CCCTCTAGA ATGAGGCCGATCCAGTGGGTTCGA | Full-length primer of GE001236 | In this study |
|  | GE001236-D-R | CCCAAGCTT TCAGCGCAATTCGCCCTCTTCG |  |  |
| P32 | GE001237-A-C-F | ATCTGCTGCAACTGcgtCTGCAGAGCGAAAACATCAAGC | Primer of 1510223,A-C | In this study |
|  | GE001237-A-C-R | AcgCAGTTGCAGCAGATTGTGACAACGTGCGC |  |  |
| P33 | GE001237-D-F | CCCTCTAGA ATGACGGCGGAGATGGATAATCG | Full-length primer of GE001237 | In this study |
|  | GE001237-D-R | CCCAAGCTT TCACACCCCCGGTCGCGCGGAT |  |  |
| P34 | GE001424-L-F | CCCGGATCC CTGTTGCGCTAGGCTGACCATG | Mutant of GE001424 | In this study |
|  | GE001424-L-R | CCCTCTAGA GGACACTACGCCGCCAGTCACG |  |  |
| P35 | GE001424-R-F | CCCTCTAGAGCAAGGCGGGTGGCAAGCTGTTGAA |  |  |
|  | GE001424-R-R | CCCAAGCTT GGGGTGTATTCGTGTACGTCGG |  |  |
| P36 | ny- GE001424-L | ATGAGATCCGTCGCTGCGTGGA | Inner primer of GE001424 | In this study |
|  | ny- GE001424-R | CCGACCTTGTTGTGCAGCTTGA |  | In this study |
| P37 | GM001327-L-F | CCCGGATCC ATGAGGTGCGGGTCATCGAGATG | Mutant of GM001327 | In this study |
|  | GM001327-L-R | CCCTCTAGA TCGAACCCACTGGATCGGCCTCA |  |  |
| P38 | GM001327-R-F | CCCTCTAGA GAGGGCGAATTGCGCTGACGTTTGC |  |  |
|  | GM001327-R-R | CCCAAGCTT CACACAATCGTCGCCGAGGAAGG |  |  |
| P39 | ny- GM001327-L | ACGAAGCCAAGAGCCGTTTCCTG | Inner primer of GM001327 | In this study |
|  | ny- GM001327-R | ATCGAACAGGCGTGTGCGCATTT |  |  |
| P40 | GE001424-L-F | CCCGGATCC TGCGAAGACCACGAAAATCCTG | Mutant of GE001424 | In this study |
|  | GE001424-L-R | CCCTCTAGA GGCGAGGCGAGGTATCGAACAT |  |  |
| P41 | GE001424-R-F | CCCTCTAGA TCTGGCAGGCGGTACAGGCTGT |  |  |
|  | GE001424-R-R | CCCAAGCTT ACCGGGTCGATCCGCTGCAATT |  |  |
| P42 | ny- GE001424-L | GACCTGCTCGATATCGGTGGCGAA | Inner primer of GE001424 | In this study |
|  | ny- GE001424-R | CGATGCTGCGCTTACGCGACAA |  |  |
| P43 | GE000287-L-F | CCCGGATCC ATCCGAGTCATGCGCTGTTCGAA | Mutant of GE000287 | In this study |
|  | GE000287-L-R | CCCTCTAGA CGCGATGACGGCGGTAATGCCTT |  |  |
| P44 | GE000287-R-F | CCCTCTAGAGCGGAGCGTAATCGCATGAGATTTT |  |  |
|  | GE000287-R-R | CCCAAGCTTCCCAGATTGTTGTCGAAAACGGT |  |  |
| P45 | ny- GE000287-L | GCATCCGTATGCGCCGAAAATCA | Inner primer of GE000287 | In this study |
|  | ny- GE000287-R | TCCTTGTACAGCGTGGAGGCGAT |  |  |
| P46 | GE000279-L-F | \| CCCGGATCC GCACACTCCCGACCTGATCAAAT \| \| --- \| | Mutant of GE000279 | In this study |
|  | GE000279-L-R | CCCTCTAGA CGTAAGCCGTCGCCACAAACCCT |  |  |
| P47 | GE000279-R-F | CCCTCTAGA AATGTCAATTACATTCAGGGATCAG |  |  |
|  | GE000279-R-R | CCCAAGCTT GCATCTCATAACACTACTTCAGA |  |  |
| P48 | ny- GE000279-L | GGCAATCAAACGCTTTCCGGCTA | Inner primer of GE000279 | In this study |
|  | ny- GE000279-R | CAATAGGCGGCGTTTGTTGTAGG |  |  |
| P49 | GE001236-L-F | CCCGGATCC TGTTCATCAATGGCAAGGAAAT | Mutant of GE001236 (rpfG) | In this study |
|  | GE001236-L-R | CCCTCTAGA ATTATCCATCTCCGCCGTCATC |  |  |
| P50 | GE001236-R-F | CCCTCTAGA TCGCGAGCAACTGCAGGTGGTCT |  |  |
|  | GE001236-R-R | CCCAAGCTT AATAAGGCGTCACGAGGATCGT |  |  |
| P51 | ny- GE001236-L | GAATTGCGCGCACGTTGTCACA | Inner primer of GE001236 | In this study |
|  | ny- GE001236-R | AATCGCTTCGCCGACCAATCCA |  |  |
| P52 | rpfC-L-F | CCCGGATCC ATGAGGTGCGGGTCATCGAGATG | Mutant of RpfC | In this study |
|  | rpfC-L-R | CCCTCTAGA TCGAACCCACTGGATCGGCCTCA |  |  |
| P53 | rpfC-R-F | CCCTCTAGGAGGGCGAATTGCGCTGACGTTTGC |  |  |
|  | rpfC-R-R | CCCAAGCTT CACACAATCGTCGCCGAGGAAGG |  |  |
| P54 | ny-rpfC-L | ACGAAGCCAAGAGCCGTTTCCTG | Inner primer of RpfC | In this study |
|  | ny-rpfC-R | ATCGAACAGGCGTGTGCGCATTT |  |  |
| P87 | RpfH-L-F | GTGAGGGCGGTGCTCAGCCAGCTGC | Primer of rpfH | In this study |
|  | RpfH-L-R | TCAGACGCGCGGCGGCGTCGATG |  |  |
