# Supplementary material for: Comparative genome analysis unravels pathogenicity of Xanthomonas albilineans causing sugarcane leaf scald disease
Source: BMC Genomics. 2022 Sep 26;23:671. doi: 10.1186/s12864-022-08900-2 (PMC9513982; doi:10.1186/s12864-022-08900-2)
Supplement: Supplementary file 3 — Additional file 3. [file 12864_2022_8900_MOESM3_ESM.zip › Table S2.docx]

**Table S2. Repeat contents from genome sequence of *Xal* JG43 *and Xsa* DD13.**

| **Type** | ***X. albilineans* JG43** | | | ***X. sacchari* DD13** | | |
| --- | --- | --- | --- | --- | --- | --- |
|  | **Number** | **Length (bp)** | **Percent (%)** | **Number** | **Length (bp)** | **Percent (%)** |
| DNA elements | - | - | - | 1 | 96 | 0.002 |
| SINEs | - | - | - | 17 | 1,290 | 0.03 |
| LINEs | - | - | - | 4 | 282 | 0.006 |
| Small RNA | - | - | - | 15 | 3,097 | 0.06 |
| Low complexity | 14 | 638 | 0.02 | 66 | 3,154 | 0.07 |
| Simple repeats | 482 | 22,068 | 0.58 | 2,531 | 138,088 | 2.82 |
| Unclassified | 2 | 274 | 0.01 | - | - | - |
| Total | 498 | 22,980 | 0.61 | 2,634 | 146,007 | 2.99 |
