# Supplementary material for: Comparative genome analysis unravels pathogenicity of Xanthomonas albilineans causing sugarcane leaf scald disease
Source: BMC Genomics. 2022 Sep 26;23:671. doi: 10.1186/s12864-022-08900-2 (PMC9513982; doi:10.1186/s12864-022-08900-2)
Supplement: Supplementary file 3 — Additional file 3. [file 12864_2022_8900_MOESM3_ESM.zip › Table S4.docx]

**Table S4. Enriched Carbohydrate-active enzymes (CAZys) in *Xal JG43 and Xsa DD13.***

| **Species** | **Carbohydrate-active enzymes** | | | | | | |
| --- | --- | --- | --- | --- | --- | --- | --- |
|  | **AA** | **CE** | **GH** | **GT** | **PL** | **CBM** | **Total** |
| *Xal* JG43 | 8 | 29 | 63 | 33 | 1 | 20 | 154 |
| *Xsa* DD13 | 12 | 52 | 95 | 50 | 5 | 28 | 242 |

Note: AA-auxiliary activity enzymes, CE-carbohydrate esterase, GH-glycoside hydrolase, GT-glycosyl transferase, PL-polysaccharide lyase, CBM-carbohydrate binding module.
