# Supplementary material for: Comparative genome analysis unravels pathogenicity of Xanthomonas albilineans causing sugarcane leaf scald disease
Source: BMC Genomics. 2022 Sep 26;23:671. doi: 10.1186/s12864-022-08900-2 (PMC9513982; doi:10.1186/s12864-022-08900-2)
Supplement: Supplementary file 3 — Additional file 3. [file 12864_2022_8900_MOESM3_ESM.zip › Table S7.docx]

**Table S7.** **Resequencing of 23 *Xanthomona* *albilineans* strains in this study.**

| **Strain** | **Location** | **Coordinate** | **Clean Reads** | **Clean Base (bp)** | **Coverage (×)** | **Mapped (%)** | **Q30(%)** | **GC (%)** |
| --- | --- | --- | --- | --- | --- | --- | --- | --- |
| JG15 | Jinguang, China | 107.93°E,22.82°N | 5,678,059 | 1,703,417,700 | 441 | 98.60 | 92.56 | 63.17 |
| JG24 | Jinguang, China | 107.93°E,22.82°N | 6,520,538 | 1,956,161,400 | 473 | 92.74 | 90.19 | 62.03 |
| JG36 | Jinguang, China | 107.93°E,22.82°N | 5,909,058 | 1,772,717,400 | 435 | 93.96 | 90.06 | 62.08 |
| JG37 | Jinguang, China | 107.93°E,22.82°N | 5,950,611 | 1,797,084,522 | 444 | 94.35 | 89.67 | 62.37 |
| NM10 | Ningming,China | 106.85°E, 22.34°N | 5,167,944 | 1,550,383,200 | 394 | 96.17 | 91.19 | 62.89 |
| NM2 | Ningming,China | 106.85°E, 22.34°N | 5,364,079 | 1,609,223,700 | 406 | 96.04 | 90.95 | 62.56 |
| FS3 | Fusui, China | 107.54°E, 22.38°N | 4,009,164 | 1,201,907,794 | 316 | 98.49 | 95.29 | 63.19 |
| FS5 | Fusui, China | 107.54°E, 22.38°N | 3,605,535 | 1,080,738,610 | 257 | 93.82 | 95.36 | 62.33 |
| FS7 | Fusui, China | 107.54°E, 22.38°N | 3,698,307 | 1,108,741,768 | 299 | 96.38 | 95.31 | 62.92 |
| FS12 | Fusui, China | 107.54°E, 22.38°N | 3,731,663 | 1,118,652,290 | 300 | 96.24 | 95.45 | 62.78 |
| FS15 | Fusui, China | 107.54°E, 22.38°N | 3,737,782 | 1,120,369,032 | 300 | 96.16 | 95.02 | 62.68 |
| FS25 | Fusui, China | 107.54°E, 22.38°N | 3,838,110 | 1,150,655,070 | 278 | 94.22 | 95.28 | 62.79 |
| FS28 | Fusui, China | 107.54°E, 22.38°N | 4,198,144 | 1,258,585,264 | 339 | 96.42 | 94.97 | 62.95 |
| FS29 | Fusui, China | 107.54°E, 22.38°N | 3,931,188 | 1,178,555,002 | 303 | 97.57 | 95.44 | 62.82 |
| FS32 | Fusui, China | 107.54°E, 22.38°N | 4,664,108 | 1,398,249,666 | 366 | 99.85 | 95.01 | 62.65 |
| FS35 | Fusui, China | 107.54°E, 22.38°N | 4,155,079 | 1,245,696,760 | 304 | 94.84 | 94.71 | 62.78 |
| FS42 | Fusui, China | 107.54°E, 22.38°N | 3,878,063 | 1,162,653,612 | 300 | 97.17 | 94.69 | 62.84 |
| FS46 | Fusui, China | 107.54°E, 22.38°N | 3,613,889 | 1,083,471,728 | 267 | 94.99 | 94.79 | 62.83 |
| FS53 | Fusui, China | 107.54°E, 22.38°N | 3,571,454 | 1,070,602,716 | 267 | 96.39 | 94.58 | 62.44 |
| FS60 | Fusui, China | 107.54°E, 22.38°N | 3,946,687 | 1,183,223,100 | 303 | 97.64 | 95.00 | 62.86 |
| FS61 | Fusui, China | 107.54°E, 22.38°N | 4,194,020 | 1,257,408,696 | 292 | 90.19 | 94.36 | 62.75 |
| FS62 | Fusui, China | 107.54°E, 22.38°N | 4,363,137 | 1,308,035,380 | 318 | 94.22 | 94.82 | 62.69 |
| FS63 | Fusui, China | 107.54°E, 22.38°N | 4,429,286 | 1,327,913,588 | 349 | 99.87 | 94.90 | 62.76 |
