# Supplementary material for: Comparative genome analysis unravels pathogenicity of Xanthomonas albilineans causing sugarcane leaf scald disease
Source: BMC Genomics. 2022 Sep 26;23:671. doi: 10.1186/s12864-022-08900-2 (PMC9513982; doi:10.1186/s12864-022-08900-2)
Supplement: Supplementary file 3 — Additional file 3. [file 12864_2022_8900_MOESM3_ESM.zip › Table S8.docx]

**Table S8. Genomic variations (SNPs and SVs) obtained from 23 sequenced strains against JG43.**

| Strains | SNPs | | | | | SVs | | | | | |
| --- | --- | --- | --- | --- | --- | --- | --- | --- | --- | --- | --- |
|  | Total | Transition | Transversion | Ti/Tv | Homozygosity | INS | DEL | INV | ITX | UN | Total |
| JG15 | 17 | 10 | 7 | 1.42 | 17 | 5 | 11 | 28 | 1,565 | 0 | 1,609 |
| JG24 | 17 | 10 | 7 | 1.42 | 17 | 0 | 3 | 1 | 1,518 | 0 | 1,522 |
| JG36 | 12 | 6 | 6 | 1 | 12 | 0 | 2 | 2 | 3,300 | 0 | 3,304 |
| JG37 | 18 | 11 | 7 | 1.57 | 18 | 20 | 2 | 21 | 698 | 0 | 741 |
| NM10 | 18 | 10 | 8 | 1.25 | 18 | 34 | 6 | 24 | 728 | 0 | 792 |
| NM2 | 17 | 12 | 5 | 2.4 | 17 | 0 | 2 | 15 | 1,713 | 0 | 1,730 |
| FS3 | 19 | 13 | 6 | 2.16 | 19 | 6 | 64 | 35 | 51 | 1 | 157 |
| FS5 | 22 | 11 | 11 | 1 | 22 | 39 | 63 | 102 | 131 | 1 | 336 |
| FS7 | 17,275 | 12,623 | 4,652 | 2.71 | 17,275 | 8 | 62 | 41 | 51 | 4 | 166 |
| FS12 | 17,274 | 12,625 | 4,649 | 2.71 | 17,274 | 18 | 61 | 52 | 70 | 1 | 202 |
| FS15 | 17,279 | 12,631 | 4,648 | 2.71 | 17,279 | 0 | 82 | 71 | 104 | 2 | 259 |
| FS25 | 20 | 10 | 10 | 1 | 20 | 4 | 52 | 23 | 32 | 0 | 111 |
| FS28 | 17,282 | 12,629 | 4,653 | 2.71 | 17,282 | 11 | 74 | 38 | 74 | 3 | 200 |
| FS29 | 15 | 9 | 6 | 1.5 | 15 | 3 | 71 | 25 | 34 | 0 | 133 |
| FS32 | 16 | 6 | 10 | 0.6 | 16 | 1 | 38 | 76 | 81 | 0 | 196 |
| FS35 | 11 | 8 | 3 | 2.66 | 11 | 2 | 43 | 53 | 47 | 0 | 145 |
| FS42 | 22 | 11 | 11 | 1 | 22 | 4 | 49 | 25 | 32 | 1 | 111 |
| FS46 | 15 | 9 | 6 | 1.5 | 15 | 4 | 31 | 9 | 19 | 0 | 63 |
| FS53 | 19 | 9 | 10 | 0.9 | 19 | 9 | 52 | 110 | 108 | 0 | 279 |
| FS60 | 24 | 12 | 12 | 1 | 24 | 2 | 47 | 17 | 28 | 0 | 94 |
| FS61 | 18 | 11 | 7 | 1.57 | 18 | 8 | 35 | 26 | 28 | 0 | 97 |
| FS62 | 29 | 16 | 13 | 1.23 | 29 | 17 | 48 | 23 | 46 | 0 | 134 |
| FS63 | 22 | 11 | 11 | 1 | 22 | 0 | 86 | 19 | 37 | 0 | 142 |

*Xal* JG43 was used as the reference genome. Transition: A/G and T/C; Transversion: A/C, A/T, G/C and G/T; Ti/TV (%): Ratio of transition to transversion.

SV: [structural](javascript:;) [variation](javascript:;); INS: Insertion; DEL: Deletion; INV: Inversion; ITX: Intra-chromosomal Translocation; UN: undermined.
