# Supplementary material for: Comparative genome analysis unravels pathogenicity of Xanthomonas albilineans causing sugarcane leaf scald disease
Source: BMC Genomics. 2022 Sep 26;23:671. doi: 10.1186/s12864-022-08900-2 (PMC9513982; doi:10.1186/s12864-022-08900-2)
Supplement: Supplementary file 3 — Additional file 3. [file 12864_2022_8900_MOESM3_ESM.zip › Table S9.docx]

**Table S9. SNP mutations in 23 strains of *Xanthomonas* species*.***

| **Strain** | **Types of SNP mutation** | | | | | |
| --- | --- | --- | --- | --- | --- | --- |
|  | **C:G>A:T** | **C:G>T:A** | **T:A>C:G** | **T:A>G:C** | **C:G>G:C** | **T:A>A:T** |
| JG15 | 3 | 3 | 7 | 2 | 2 | 0 |
| JG24 | 4 | 4 | 6 | 1 | 2 | 0 |
| JG36 | 4 | 2 | 4 | 1 | 1 | 0 |
| JG37 | 4 | 4 | 7 | 1 | 2 | 0 |
| NM10 | 3 | 4 | 6 | 2 | 2 | 1 |
| NM2 | 3 | 4 | 8 | 1 | 1 | 0 |
| FS3 | 1 | 6 | 7 | 1 | 4 | 0 |
| FS5 | 3 | 4 | 7 | 1 | 6 | 1 |
| FS7 | 1268 | 6360 | 6261 | 1286 | 1790 | 307 |
| FS12 | 1266 | 6361 | 6263 | 1284 | 1790 | 307 |
| FS15 | 1266 | 6361 | 6268 | 1286 | 1788 | 307 |
| FS25 | 5 | 4 | 6 | 1 | 4 | 0 |
| FS28 | 1262 | 6359 | 6268 | 1290 | 1792 | 308 |
| FS29 | 1 | 3 | 6 | 1 | 4 | 0 |
| FS32 | 5 | 3 | 3 | 1 | 4 | 0 |
| FS35 | 1 | 4 | 4 | 1 | 1 | 0 |
| FS42 | 5 | 4 | 7 | 1 | 4 | 1 |
| FS46 | 1 | 3 | 6 | 1 | 4 | 0 |
| FS53 | 4 | 4 | 5 | 1 | 4 | 1 |
| FS60 | 5 | 5 | 7 | 1 | 5 | 1 |
| FS61 | 2 | 7 | 4 | 1 | 4 | 0 |
| FS62 | 4 | 7 | 9 | 2 | 6 | 1 |
| FS63 | 6 | 3 | 8 | 1 | 3 | 1 |

Note: Genome-wide SNP mutations can be divided into six categories. T:A>C:G mutations include T>C and A>G. When T>C type mutations appear in the positive chain of the reference genome, A>G type mutations are in the same position of the negative chain of the reference genome, T>C and A>G are divided into a class.
