# Supplementary material for: Comparative genome analysis unravels pathogenicity of Xanthomonas albilineans causing sugarcane leaf scald disease
Source: BMC Genomics. 2022 Sep 26;23:671. doi: 10.1186/s12864-022-08900-2 (PMC9513982; doi:10.1186/s12864-022-08900-2)
Supplement: Supplementary file 3 — Additional file 3. [file 12864_2022_8900_MOESM3_ESM.zip › Table S1.doc]

**Table S1. Basic information of *Xal* JG43 and *Xsa* DD13.**

| Strain | *Xal* JG43 | *Xsa* DD13 |
| --- | --- | --- |
| **Species** | *X. albilineans* | *X. sacchari* |
| **Collected sites** | Jinguang, China | Dingdang, China |
| **Coordinate** | 107.93°E,22.82°N | 108.86°E,23.34°N |
| **Collected hosts** | GT46 | ROC22 |
| **Disease** | Sugarcane leaf scald | Sugarcane leaf chlorotic streak disease |
| **Colony characters** | Circular, convex, smooth, shiny, yellow pigmented bacterial colonies | Small, circular, convex, glossy, translucent, non-mucoid, and faintly yellow bacterial colonies. Bacteria were bullet-shaped, with single, polar flagella |
| **Disease symptoms** | White stripes along veins; shorter stalk internodes, smaller leaves, and abnormal development of side shoots along the stalk; a sudden wilting of all leaves. | Chlorotic white streak symptoms (“pencil lines”) that extended longitudinally from the base to the tip of leaves. Wilting of the plants rapidly followed. |
